# Parameterizing steady-state C_3_ photosynthesis models using photosynthesis response curves: which models to use and which parameter estimates to trust?

**DOI:** 10.1093/jxb/erag340

**Published:** 2026-07-17

**Authors:** Rudan Xu, John N Ferguson, Salma Tariq, Patience B Iliya, Anika Küken, Johannes Kromdijk, Zoran Nikoloski

**Affiliations:** Bioinformatics Department, Institute of Biochemistry and Biology, University of Potsdam, Potsdam, Germany; Systems Biology and Mathematical Modelling Group, Max Planck Institute of Molecular Plant Physiology, Potsdam, Germany; School of Life Sciences, University of Essex, Colchester, UK; Bioinformatics Department, Institute of Biochemistry and Biology, University of Potsdam, Potsdam, Germany; Bioinformatics Department, Institute of Biochemistry and Biology, University of Potsdam, Potsdam, Germany; Systems Biology and Mathematical Modelling Group, Max Planck Institute of Molecular Plant Physiology, Potsdam, Germany; Bioinformatics Department, Institute of Biochemistry and Biology, University of Potsdam, Potsdam, Germany; Department of Plant Sciences, University of Cambridge, Cambridge, UK; Bioinformatics Department, Institute of Biochemistry and Biology, University of Potsdam, Potsdam, Germany; Systems Biology and Mathematical Modelling Group, Max Planck Institute of Molecular Plant Physiology, Potsdam, Germany; Stellenbosch University, South Africa

**Keywords:** C_3_ photosynthesis, FvCB model variants, gas exchange measurements, parameter estimation, steady-state modeling, uncertainty analysis

## Abstract

Photosynthesis sustains life on Earth, yet we still lack comprehensive understanding of the biochemical and environmental factors that affect this fundamental process. Steady-state models of C_3_ photosynthesis provide a powerful framework but rely on reliable estimation of numerous parameters from gas exchange data. Despite advances, how model structure and data choice influence parameter accuracy and consistency remains poorly explored. Here, we systematically evaluate parameterization across nine steady-state photosynthesis models and different levels of gas exchange measurements. Using synthetic photosynthesis response curves generated from the examined models with sampled parameter values, we applied Bayesian inference to quantify parameter uncertainty and estimation of performance for the considered models. We showed that while key parameters of C_3_ photosynthesis can be reliably estimated from a single *A–C*_i_ curve, others require expanded sampling of light response space. We also demonstrated the advantage of using simultaneous estimation of all model parameters over biasing the estimation by keeping some parameters fixed to prior values. Barley gas exchange data further demonstrated that parameter consistency across models can be evaluated comparing different levels of measurements and depends strongly on both model formulation and data type. Together, our study provides practical guidance for selecting photosynthesis models, designing phenotyping strategies, and choosing parameterization approaches for steady-state C_3_ photosynthesis.

## Teaser

This study provides practical guidance on which steady-state C_3_ photosynthesis models and response curve measurements allow reliable estimation of key photosynthetic parameters from gas exchange data.

## Introduction

Photosynthesis is the fundamental biochemical process that converts light energy and atmospheric CO_2_ into sugars to sustain growth and other processes in all photosynthetic organisms. Although the biochemical and physiological processes underlying photosynthesis have been extensively studied, understanding how these processes interact to determine photosynthetic performance under different environmental conditions remains a major challenge (Kaiser *et al.*, 2015). Models of photosynthesis provide a powerful framework for integrating current knowledge and quantifying the contribution of different processes to photosynthetic function (Stirbet *et al.*, 2024). Such quantification depends on the accurate estimation of model parameters from experimental data, as these parameters, such as the maximum rate of ribulose bisphosphate carboxylase (RuBP)-saturated carboxylation (*V*_cmax_), leaf mitochondrial respiration (*R*_d_), and maximum rate of electron transport (ET) through PSII, denoted *J*_max_, capture key characteristics. Reliable parameter values are crucial not only for generating reliable model predictions for different plant genotypes and environments, but also for guiding breeding efforts toward improved photosynthetic efficiency (Long *et al.*, 2025). Therefore, improving parameter estimation remains an important prerequisite for the use of photosynthesis models in generating hypotheses and predictions across diverse plant species.

The Farquhar–von Caemmerer–Berry (FvCB) model has provided the foundation for steady-state modeling of C_3_ photosynthesis (Farquhar *et al.*, 1980). Since its development, the FvCB model has been applied in leaf-level physiology and ecological studies (Collatz *et al.*, 1991; De Pury and Farquhar, 1997). Later it was incorporated into some crop growth models, facilitating key insights into how photosynthesis is linked to the development and phenology of agriculturally relevant crops (Di Paola *et al.*, 2016). The FvCB model considers that the carboxylation rate is either limited by Rubisco activity or by RuBP regeneration, which depends on the energy supplied by the light reactions. Later variants have incorporated additional processes, including: alternative electron transport (Yin *et al.*, 2004), mesophyll conductance (*g*_m_) (Harley *et al.*, 1992; Ethier and Livingston, 2004; Price *et al.*, 2011; Tholen *et al.*, 2012), and triose phosphate utilization (TPU) limitation (Sharkey, 1985; Von Caemmerer, 2000; Busch *et al.*, 2018; Busch, 2020).

Parameter estimation relies on integrating data related to photosynthesis into models of photosynthesis. To this end, advances in gas exchange measurements have facilitated the quantification of the net CO_2_ assimilation rate, denoted by *A* or *A*_n_, across a range of intracellular CO_2_ concentration (*C*_i_) and incident light intensity (*Q*, i.e. photosynthetic photon flux density, PPFD), resulting in the characteristic *A–C*_i_ and *A–Q* response curves, respectively. Complementary chlorophyll fluorescence measurements allow the estimation of ET efficiency through PSII. Together, these response curves comprise key experimental data facilitating the investigation of *in vivo* biochemical and biophysical limitations of photosynthesis (Long and Bernacchi, 2003; Busch *et al.*, 2024).

Early studies on parameter estimation in steady-state models of photosynthesis relied on disjunct segment estimation approaches, where the *A–C*_i_ curve was divided into distinct limitation phases, and parameters were extracted separately, as reviewed in Miao *et al.* (2009). Later studies highlighted the advantage of fitting the entire *A–C*_i_ curve simultaneously, which improved estimation stability and global accuracy (Dubois *et al.*, 2007; Sharkey *et al.*, 2007; Patrick *et al.*, 2009; Su *et al.*, 2009; Gu *et al.*, 2010).

The focus of recent research efforts has undergone a transition toward the development of software tools that standardize the parameter estimation procedure and make them more accessible. These tools include Plantecophys (R package; Duursma, 2015), PCEcalculator (Excel; Sharkey, 2016), msuRACiFit (R shiny app; Gregory *et al.*, 2021), Photosynthesis (R package; Stinziano *et al*., 2021), PhotoGEA (R package; Lochocki *et al.*, 2025), and PhoTorch (Python package; Lei *et al.*, 2025). These tools differ in the FvCB variant implemented, the experimental data they handle, and whether parameters are fixed, user-defined, or fitted (see Supplementary Table S1 for comparison).

Different FvCB variants have been developed to address specific biological questions, and the choice of model is typically guided by the process of interest. For example, estimation of TPU requires a model formulation that explicitly includes TPU limitation, whereas resolving mesophyll diffusion necessitates models that account for finite mesophyll conductance. In such cases, parameters estimated from simplified formulations (e.g. assuming infinite mesophyll conductance) should be interpreted as apparent quantities and are not directly comparable with parameters defined at the chloroplastic level. Lochocki and McGrath (2025) further showed that, even when the same biochemical limitation rates are assumed, different mathematical formulations for computing *A*_n_, such as min-*A* versus min-*W* formulations, can lead to substantial differences in predictions and inferred parameters. They also showed that differences in the treatment of TPU limitation can introduce additional errors by altering how biochemical limitation is represented. Such formulation-dependent differences are relevant beyond individual curve fitting because leaf-level photosynthesis models and their parameterizations are used in crop, terrestrial biosphere, and Earth system models, where uncertainty in photosynthetic representation can propagate to larger-scale predictions (Rogers *et al.*, 2017; Walker *et al*., 2021).

A second important difference among these tools is the type of experimental data they use for parameter estimation. Beyond steady-state model fitting, these tools include functions to assist the estimation of individual parameters from experimental data. For example, *g*_m_, can be estimated using combined gas exchange and chlorophyll fluorescence data (Yin *et al.*, 2009, 2011; Sun *et al.*, 2014; Bellasio *et al.*, 2016) in packages such as Photosynthesis and PhotoGEA. Likewise, mitochondrial respiration, *R*_d_, can be derived from gas exchange data without fitting a steady-state model (Kok, 1956; Yin *et al.*, 2011; Walker and Ort, 2015) in the Photosynthesis package. More generally, the information available for parameter estimation depends on how *A*_n_ is measured across environmental conditions, including variation in CO_2_ concentration and irradiance. These different configurations of response curves determine which photosynthetic limitations are probed and, therefore, which model parameters can be reliably constrained.

Another important distinction among these tools is how parameters are treated during fitting. Many workflows estimate only a subset of parameters while fixing others to literature, user-defined, or default values. This strategy can reduce the dimensionality of the estimation problem and improve practical usability, but it may also affect estimation accuracy if the fixed values are inconsistent. Xiao *et al.* (2021) systematically evaluated this trade-off for a specific FvCB formulation using a Bayesian framework, showing that relaxing fixed parameter assumptions increased posterior uncertainty and estimates of key parameters, such as *V*_cmax_. They further demonstrated that more informative measurement protocols, including measurements under low CO_2_ concentration and at multiple light levels, can reduce the number of parameters that need to be fixed for reliable estimation.

Building on these advances, it remains important to evaluate how model formulation, data availability, and parameter treatment jointly influence parameter identifiability and estimation robustness under a common inference framework. To address this need, we applied Bayesian inference to assess parameter identifiability and estimation performance using both synthetic and real gas exchange data. The analyses with synthetic data allowed us to compare nine steady-state FvCB model variants under controlled conditions, where the true parameter values were known, and to test how estimation accuracy changed across single *A–C*_i_ curves, combined *A–C*_i_ and *A–Q* curves, and multi-light *A–C*i curves. We further compared fitting strategies in which all parameters were estimated simultaneously with scenarios in which only core parameters were estimated while non-core parameters were fixed. Finally, using gas exchange measurements collected from barley plants grown under field conditions, we evaluated whether parameter estimates remained consistent across different levels of data aggregation. Together, these analyses provide a unified framework for assessing which parameters can be estimated with a given dataset and which model formulations are suitable for reliable estimation of particular parameters. Therefore, our findings offer practical guidance for model selection and experimental design in applications that rely on parameter estimation from steady-state models of C_3_ photosynthesis.

## Materials and methods

### Computational methods

#### Collection of FvCB models

Nine mechanistic models derived from the original FvCB model formulation (Farquhar *et al.*, 1980) were collected and implemented for comparative analysis. These models represent key developments in photosynthetic modeling over the past four decades, each incorporating additional biochemical or biophysical processes beyond the canonical formulation. For the purpose of describing model selection, we first introduce the selected variants in approximate chronological order, reflecting the historical development of the FvCB framework.

The first model, FvCB80, corresponds to the canonical version (Farquhar *et al.*, 1980). In this formulation, the minimum of the Rubisco-limited carboxylation rate (*W*_c_) and RuBP regeneration-limited carboxylation rate (*W*_j_) is first determined, giving the limiting carboxylation rate (*W*_m_). *A*_n_ is then calculated from this limiting rate and the formulation is therefore referred to as a min-*W* formulation.

The second model, Harley92, extends the canonical formulation by incorporating *g*_m_, allowing for the explicit calculation of chloroplastic CO_2_ concentration (*C*_c_) (Harley *et al.*, 1992). In an explicit method, *C*_c_ is solved from the balance equation between *C*_i_ and *g*_m_, which yields a quadratic form depending on the chosen representation of *g*_m_. In contrast, when the measured net assimilation rate (*A*_n_) is used to estimate *C*_c_ through the same relationship, the approach becomes implicit. Several estimation strategies for *g*_m_ were discussed in the study (Harley *et al.*, 1992).

The third model, *v*onC00 (Von Caemmerer, 2000), is one of the most widely adopted FvCB variants. It introduces an additional limitation by TPU and considers the fraction of glycerate carbon exported from the Calvin–Benson cycle. The fourth model, Ethier04 (Ethier and Livingston, 2004), reformulates the *A*_n_ as an explicit quadratic function of internal CO_2_ concentrations by directly accounting for mesophyll conductance. This approach allows *A*_n_ to be solved analytically without the need to calculate *C*_c_ in advance, and allows direct estimation of *g*_m_ together with other parameters. This formulation corresponds to a min-*A* variant, in which *A*_n_ is determined by taking the minimum of the directly calculated assimilation rates rather than first by *W*_m_.

The fifth model, Yin04 (Yin *et al.*, 2004), generalizes the RuBP regeneration-limited rate by representing it as a co-limitation between NADPH and ATP supply, thereby allowing for flexibility in representing the alternative electron transport chain. The Dubois07 model (Dubois *et al.*, 2007) closely follows the vonC00 formulation, but differs in that the minimization procedure is applied at the net assimilation level rather than the carboxylation rate level; as a result, it shows slightly different sensitivity near the transition points between limitations.

In the Tholen12 model (Tholen *et al.*, 2012), mesophyll conductance is further decomposed into its biochemical (*g*_ch_) and physical (*g*_wp_) components, allowing for the separate treatment of CO_2_ diffusion through the cell wall–plasma membrane interface and the chloroplast envelope. The Busch18 model (Busch *et al.*, 2018) introduces an explicit coupling between photosynthetic carbon and nitrogen metabolism, accounting for nitrogen assimilation as a sink for reducing power and for the diversion of glycolate carbon from the photorespiratory pathway toward serine and *de novo* nitrogen synthesis.

Finally, the Xiao21 model (Xiao *et al.*, 2021) extends the Ethier04 framework to incorporate parameters retrievable from chlorophyll fluorescence data, which enrich the dataset available for model fitting and impose stronger constraints on parameter estimation.

#### Simulation of synthetic data

Synthetic datasets were generated using the implemented FvCB model variants under varying *C*_i_ concentrations and PPFDs. Specifically, *C*_i_ values ranged from 50 µmol CO_2_ mol^−1^ to 1500 µmol CO_2_ mol^−1^ (50, 100, 150, 200, 300, 500, 800, 1000, 1200, and 1500 µmol CO_2_ mol^−1^) and PPFD levels from 50 µmol m^−2^ s^−1^ to 1800 µmol m^−2^ s^−1^ (50, 150, 300, 500, 1200, and 1800 µmol m^−2^ s^−1^).

Three types of synthetic data were generated for subsequent Bayesian parameter estimation: (i) a single *A–C*_i_ curve simulated at a constant PPFD (1800 µmol m^−2^ s^−1^); (ii) a combination of one *A–C*_i_ curve at 1800 µmol m^−2^ s^−1^ PPFD and one *A–Q* (light response) curve at constant *C*_i_ = 300 µmol CO_2_ mol^−1^; and (iii) multiple *A–C*_i_ curves simulated across all PPFD levels to represent combined CO_2_–light response curves.

To ensure the generalizability of comparison between models, 100 sets of parameters were randomly sampled within a range of physiologically plausible values (see Table 1 for the parameter ranges used for sampling) from a two-sided truncated normal distribution, similar to the study of Xiao *et al*. (2021). The generated synthetic data were used as fitting data for Bayesian inference to evaluate the identifiability and estimation robustness of model parameters under different experimental designs.

**Table 1.** Description of model parameters, their definitions, and inclusion across models

Parameters	Definition	Models	Lower–upper bounds (units)
*V*_cmax_	Maximum rate of RuBP-saturated carboxylation	All nine models	50–200 (µmol m^−2^ s^−1^)
*J*_max_	Maximum rate of electron transport (ET) through PSII	All nine models	100–250 (µmol m^−2^ s^−1^)
*R*_d_	Leaf mitochondrial respiration	All nine models	0.5–5 (µmol m^−2^ s^−1^)
Γ*	CO_2_ compensation point in the absence of dark respiration	All nine models	10–60 (µmol mol^−1^)
*K*_C_	Michaelis–Menten constant for CO_2_	All nine models	200–300 (µmol mol^−1^)
*K*_O_	Michaelis–Menten constant for O_2_	All nine models	150–300 (mmol mol^−1^)
θ_J_	Convexity factor for response of electron transport rate to light	All nine models except Harley92 and Xiao21	0.4–0.9 (–)
α_J_	Quantum yield for electron flow	All nine models except Yin04 and Xiao21	0.3–0.5 (mol e^−^ mol^−1^ PPFD)
*V*_TPU_	Rate of triose phosphate utilization	vonC00, Dubois07, and Busch18	8–20 (µmol m^−2^ s^−1^)
*g*_m_	Mesophyll conductance	Harley92, Ethier04, and Xiao21	0.1–0.7 (mol m^−2^ s^−1^)
α_G_	Fraction of the glycolate carbon exported from chloroplast	vonC00, Dubois07, and Busch18	0.3–0.8 (–)
Φ_IIm_	Maximum ET efficiency of PSII	Yin04	0.5–0.9 (mol e^−^ mol^−1^ PPFD)
*f*_Q_	Fraction of ET following the Q-cycle	Yin04	0.5–1 (–)
*f*_pseudo_	Fraction of pseudocyclic ET	Yin04	0–0.3 (–)
*h*	The number of protons required to produce 1 ATP mol mol^−1^	Yin04	3–4.67 (mol mol^−1^)
*g*_ch_	Conductance through chloroplast envelope and stroma	Tholen12	0.05–0.7 (mol m^−2^ s^−1^)
*g*_wp_	Conductance through chloroplast wall and plasmalemma	Tholen12	0.1–2 (mol m^−2^ s^−1^)
max_αG_	Maximum glycolate carbon fraction exported as glycine	Busch18	0.01–0.2 (–)
max_αS_	Maximum glycolate carbon fraction exported as serine	Busch18	0.01–0.5 (–)
*N*_max_	Maximum rate of *de novo* nitrogen supply to the chloroplast	Busch18	0.1–2 (µmol m^−2^ s^−1^)
*s*	Product of light absorption and partition of total absorbed light to PSII	Xiao21	0.3–0.6 (–)
Φ_IILL_	Maximum quantum efficiency of PSII under low light	Xiao21	0.5–1 (–)

Included are the parameters present in each model, along with their corresponding lower and upper bounds (including units), used for parameter sampling and estimation.

To simulate measurement uncertainty, Gaussian noise was added to the model-simulated *A*_n_. The SD of the noise combined a constant absolute component (0.2 µmol m^−2^ s^−1^) and a relative component proportional to the predicted *A*_n_ (5%).

#### Bayesian inference

Stan, a probabilistic programming language that implements full Bayesian statistical inference via Markov chain Monte Carlo (MCMC), was used in this study. The analysis was performed using the ‘rstan’ package implemented in the R programming environment (Guo *et al.*, 2020).

For Bayesian inference, each parameter of the different models is constrained to *a priori* probability distribution (the same bounds as shown in Table 1). The resulting synthetic data (three types) were then used for model fitting.

The observed assimilation rates (*A*_obs_) were assumed to follow a normal distribution centered at the model predictions:


$$A_{\mathrm{obs}}∼N(A_{\mathrm{sim}},\sigma \;),$$

where σ denotes the residual SD, which was assigned a weakly informative half-normal prior with an SD of 2. This formulation allowed joint posterior inference of the biochemical parameters governing the photosynthetic rate, providing uncertainty estimates consistent with physiological plausibility.

#### Comparison between the estimation of all parameters and a core set of parameters

To assess estimation accuracy, we randomly sampled 100 parameter sets, which were treated as the true parameter values, and simulated three types of synthetic response curves for each set, as described in the previous section. For each of the 100 sampled parameter sets, Bayesian inference was performed separately for each synthetic data type to estimate model parameters. This yielded, for every parameter and every data type, a vector of 100 maximum *a posteriori* (MAP) estimates paired with their corresponding 100 true values. Estimation accuracy was quantified using Pearson’s correlation coefficient, *r*, between these paired vectors.

In many parameter estimation workflows, not all model parameters are estimated simultaneously; instead, a subset of parameters is estimated while the remaining parameters are fixed. To test how this practice affects the accuracy of core parameter estimation, we designed three estimation scenarios. In the first scenario, all model parameters were estimated simultaneously from each synthetic data configuration. In the second scenario, only a core subset of parameters was estimated, and these include *V*_cmax_, *J*_max_, *R*_d_, and the CO_2_ compensation point in the absence of dark respiration (Γ*). All remaining model parameters were fixed to the median of their sampled distributions within the physiological ranges listed in Table 1. This scenario mimics the common practice of using fixed literature- or population-level values for parameters that are not estimated. In the third scenario, the same core parameters were estimated, but the remaining parameters were fixed to their corresponding sampled values, which served as the true values used to generate the synthetic data. This scenario provides the upper bound estimation accuracy for the core subset.

### Experimental methods

Gas exchange measurements used in this study were obtained from a previously published field phenotyping experiment on the barley (B1K) diversity panel conducted at the National Institute of Agricultural Botany in Cambridge, UK, in 2022 (Breil-Aubert *et al.*, 2025). The original study included 270 barley accessions grown in a twice-replicated alpha-lattice common garden design, with each accession represented by two plots. Each plot consisted of four rows, with the two inner rows containing transplanted wild barley plants. Phenotyping was performed within 10 d of heading date. Three plants were phenotyped per plot, and plants from the same plot were measured on the same day. On the day before phenotyping, selected stems were excised, placed in water, returned to the laboratory, recut under water, and maintained overnight before gas exchange measurements were performed on the penultimate leaf.

*A–Q* measurements were performed using LI-6400XT infrared gas analyzers equipped with leaf chamber fluorometer LED light sources. Leaves were light-acclimated for 30 min under saturating irradiance of 1800 µmol m^−2^ s^−1^ photosynthetically active radiation (PAR). Leaf chamber conditions were set to 25 °C, with an air flow rate of 400 µmol s^−1^, 65–75% relative humidity, and a reference CO_2_ concentration of 400 µmol mol^−1^. Irradiance was then decreased stepwise at 1800, 1100, 500, 300, 150, and 50 µmol m^−2^ s^−1^ PAR, with photosynthesis logged after 2 min at each light level.

*A–C*_i_ measurements were performed using LI-6800 infrared gas analyzers. Leaf chamber conditions were set to 25 °C, an air flow rate of 400 µmol s^−1^, 65% relative humidity, an initial reference CO_2_ concentration of 400 µmol mol^−1^, and saturating irradiance of 1800 µmol m^−2^ s^−1^ PAR. Once stomatal conductance and photosynthesis had stabilized, the reference CO_2_ concentration was varied stepwise at 400, 300, 200, 100, 50, 400, 400, 700, 1000, 1300, and 1800 µmol mol^−1^, with 1.5–3 min between steps depending on standard stability criteria.

For our analysis, we used the subset of 85 genotypes for which paired *A–C*_i_ and *A–Q* curve measurements were available, comprising 354 plant-level measurements that were aggregated to 156 plot-level observations.

Parameter estimation was performed at three levels: genotype, plot, and individual leaf. For genotype-level estimation, all gas exchange data points belonging to a given genotype were used for fitting, typically including up to six individual measurements (two plots with three leaves each) across varying *C*_i_ and PPFD levels. For plot-level estimation, only the data associated with a single plot were used, while for leaf-level estimation, each individual response curve (*A–C*_i_ and/or *A–Q*) was fitted independently.

The consistency of parameter estimates across these levels was explored, and the differences across models were subsequently evaluated using genotype-level estimates. To quantify model similarity, Euclidean distances were calculated between vectors of shared parameters estimated from different models for the same dataset (e.g. at the genotype level). The mean Euclidean distance across genotypes was then computed to summarize overall model divergence, and the coefficient of variation (CV) of these distances was used to assess variability among genotypes.

The Akaike information criterion (AIC) score was calculated using the following equation:


$$\mathrm{AIC}=2k-2\ln(\hat{L}),$$

where *k* is the number of parameters in the model and $\hat{L}$ is the maximized log-likelihood from Bayesian inference.

## Results

### Comparison of boundaries and considered processes of models

From the broader set of FvCB-type formulations and related extensions described in the literature (Farquhar *et al.*, 1980; Sharkey, 1985; Collatz *et al.*, 1991; Harley and Sharkey, 1991; Harley *et al.*, 1992; Ögren and Evans, 1993; Von Caemmerer, 2000; Ethier and Livingston, 2004; Yin *et al.*, 2004; Dubois *et al.*, 2007; Price *et al.*, 2011; Tholen *et al.*, 2012; Evans and Von Caemmerer, 2013; Moualeu-Ngangue *et al.*, 2017; Busch *et al.*, 2018; Busch, 2020; Xiao *et al.*, 2021), as summarized in previous reviews (Bernacchi *et al.*, 2013; Von Caemmerer, 2013; Yin *et al.*, 2021), we selected nine models for detailed analysis. For clarity, each model is referred to by the first author’s name and publication year. The selection was designed to represent the main structural extensions of the original FvCB framework rather than to provide an exhaustive implementation of all published models, as model formulation differences determine which processes are represented explicitly and which parameters could be estimated or should be fixed from experimental data.

To interpret differences among simulated *A–C*_i_ curves, we classified the selected models according to their treatment of mesophyll CO_2_ diffusion, the inclusion of TPU limitation, and alternative ET formulation. Specifically, we summarized how each model inherits or modifies the main limitation terms of the original FvCB80 formulation (see Fig. 1A).

**Fig. 1.**
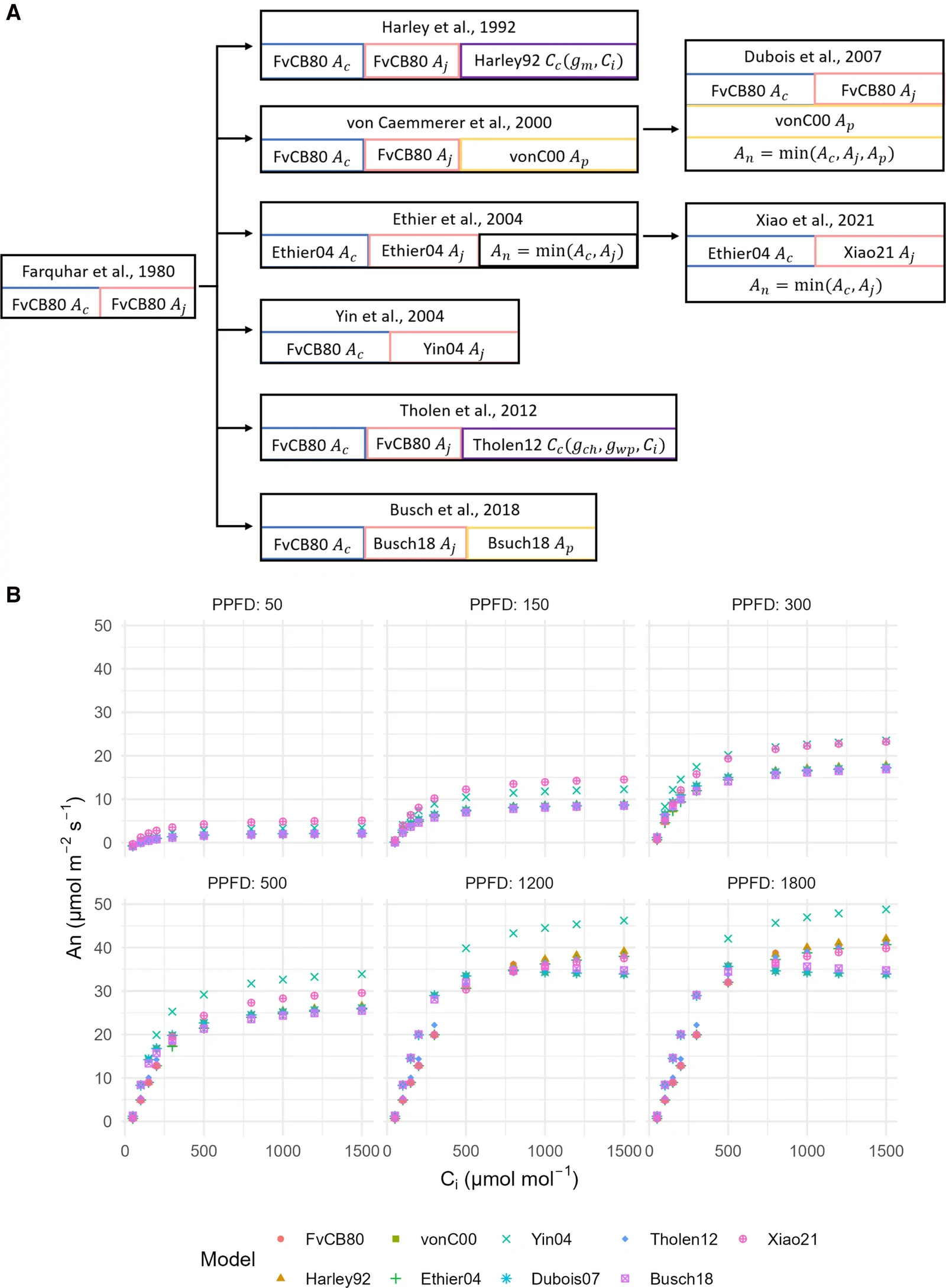
Overview of the kinship between FvCB models and their effects on simulated *A–C*_i_ curves. (A) Schematic representation of the relationships among the nine FvCB model variants included in this study. Arrows indicate inheritance or modification of model components from earlier formulations, tracing the lineage from the original FvCB formulation (Farquhar *et al.*, 1980). The labelled compartments within each model box denote key variants representing different biochemical formulations of photosynthetic limitations: Rubisco (*A*_c_), RuBP regeneration (*A*_j_), TPU (*A*_p_), mesophyll conductance (*g*_m_). (B) Simulated *A–C*_i_ response curves across multiple light levels (PPFD = 50–1800 µmol m^−2^ s^−1^) generated using each model with the same values for the set of shared parameters.

The first group comprises models that modify the treatment of CO_2_ diffusion between the intercellular airspace and the chloroplast; the Harley92 model (Harley *et al.*, 1992) was one of the first to explicitly integrate finite *g*_m_ into the FvCB framework by replacing *C*_i_ with *C*_c_ in the biochemical limitation terms. The Tholen12 model further extends this representation by partitioning *g*_m_ into biochemical and physical components (Tholen *et al.*, 2012). Both Harley92 and Tholen12 models retain the min-*W* structure of the original FvCB80 model. In contrast, the Ethier04 model reformulated both *A*_c_ and *A*_j_ as explicit quadratic equations that account for *g*_m_ and *C*_i_, and allow min-*A* formulation.

The second group comprises models that extend the biochemical limitation structure through the inclusion of TPU limitation. The vonC00 model extends the min-*W* formulation of the FvCB80 model by including the TPU-limited carboxylation rate (*W*_p_), following Harley and Sharkey (1991). The Dubois07 model uses the same *W*_c_, *W*_j_, and *W*_p_ formulations as the vonC00 model, but it calculates *A*_n_ as the minimum among *A*_c_, *A*_j_, and *A*_p_. The Busch18 model retains the min-*W* structure from the vonC00 model while further modifying the *W*_j_ and *W*_p_ components.

Within the third group modifying the ET formulation, the Yin04 model keeps the *W*_c_ formulation from the FvCB80 model but modifies the corresponding *W*_j_ by incorporating alternative ET pathways. The Xiao21 model overlaps with both the *g*_m_ and ET extension classes: it inherits the finite *g*_m_ and min-*A* structure from the Ethier04 model and further modified *A*_j_ by introducing additional parameters such as the product of light absorption and partition of total absorbed light to PSII, *s*, and the maximum quantum efficiency of PSII under low light (Φ_IILL_).

All models use the same Rubisco-limited rate, *W*_c_, and formulation, but differ in whether *C*_c_ or *C*_i_ is used as the CO_2_ concentration. Consequently, all model variants rely on a set of parameters (Table 1), including *V*_cmax_, *J*_max_, *R*_d_, Γ*, the Michaelis–Menten constant for CO_2_ (*K*_C_), the Michaelis–Menten constant for O_2_ (*K*_O_), the convexity factor for response of ET rate to light (θ_J_), and quantum yield for electron flow (α_J_), with the last two parameters excluded in some models depending on the specific ET formulation. This distinction leads to the differences in the initial slope of simulated *A–C*_i_ curves, although the values for the selected parameters were identical in the simulations (Fig. 1B).

Models considering a finite mesophyll conductance, *g*_m_, namely the Harley92, Ethier04, Tholen12, and Xiao21 models, exhibited a slower increase in *A*_n_ at low *C*_i_ concentrations compared with other models, namely the FvCB80, vonC00, Yin04, Dubois07, and Busch18, that assume infinite *g*_m_. In the Yin04 model, the parameters representing alternative ET, such as fraction of ET into the Q-cycle, *f*_Q_, and the fraction of the pseudocyclic ET (*f*_pseudo_; see Table 1), were set to zero. Like in the FvCB80 model, the number of protons required to produce ATP, denoted by *h*, was set to 3 and the fraction of the cyclic ET around PSI, *f*_cyc_, was computed accordingly, resulting in higher *A*_n_ values under high light intensities (PPFD from 500 µmol m^−2^ s^−1^ to 1800 µmol m^−2^ s^−1^). Under higher light conditions (e.g. 1200 µmol m^−2^ s^−1^ and 1800 µmol m^−2^ s^−1^ PPFD), models that include TPU limitation, namely the vonC00, Dubois07, and Busch18 models, reached a lower plateau at high environmental CO_2_ concentrations than models without TPU limitation (Fig. 1B). Thus, differences in simulated *A*_n_ originated from model structure rather than differences in the values of shared parameters, indicating that distinct model formulations can yield different outputs even under the same parameterization.

### Parameter uncertainty across different models and response curves

To evaluate parameter estimation uncertainty across the nine FvCB model variants, we randomly generated 100 parameter sets by sampling from a two-sided truncated normal distribution (Supplementary Fig. S1A). These sampled values were treated as ground-truth parameters and used to create three types of synthetic data for each model: (i) a single *A–C*_i_ curve simulated at saturating light condition (PPFD = 1800 µmol m^−2^ s^−1^); (ii) a combination of *A–C*_i_ (at saturating light) and *A–Q* curves (at *C*_i_ = 300 µmol CO_2_ mol^−1^), and (iii) *A–C*_i_ curves simulated at multiple light levels (PPFD = 50, 150, 300, 500, 1200, and 1800 µmol m^−2^ s^−1^), referred to as multi-light *A–C*_i_ curves. A summary of the synthetic *A–C*_i_ curves at saturating light is shown in Supplementary Fig. S1B (see the Materials and methods for details).

Mathematically, a parameter is considered identifiable for a given dataset if its value can be uniquely determined from the data. Here, we take a more lenient approach and quantify parameter uncertainty using the 50% highest posterior density interval (Press, 2009) as the credible interval and calculate its width relative to the sampling bounds. An increase in the relative credible interval (RCI) width reflects reduced precision and, therefore, greater uncertainty in the inferred parameter value.

Among the key parameters, *V*_cmax_, *J*_max_, and Γ* exhibited smaller RCI width when fitted with a single *A–C*_i_ curve (∼20%; Fig. 2), although Γ* showed greater variability across the 100 repetitions. Differences between models were also observed; for example, *V*_cmax_ estimated from the Tholen12 and Xiao21 models exhibited broader RCI widths compared with other models. When *A–C*_i_ curves at multiple PPFD levels (multi-light *A–C*_i_ curves) were fitted, the uncertainty for *V*_cmax_, *J*_max_, and Γ* further decreased. In contrast, the RCI width of *R*_d_ showed moderate uncertainty (∼40%) under single *A–C*_i_ fitting, but it decreased to <20% when multi-light *A–C*_i_ curves were used. Likewise, θ_J_ and α_J_ displayed high uncertainty (RCI width ∼60%; Supplementary Fig. S2A) when only a single *A–C*_i_ curve was fitted, yet achieved substantially narrower RCI widths when multi-light *A–C*_i_ curves were included.

**Fig. 2.**
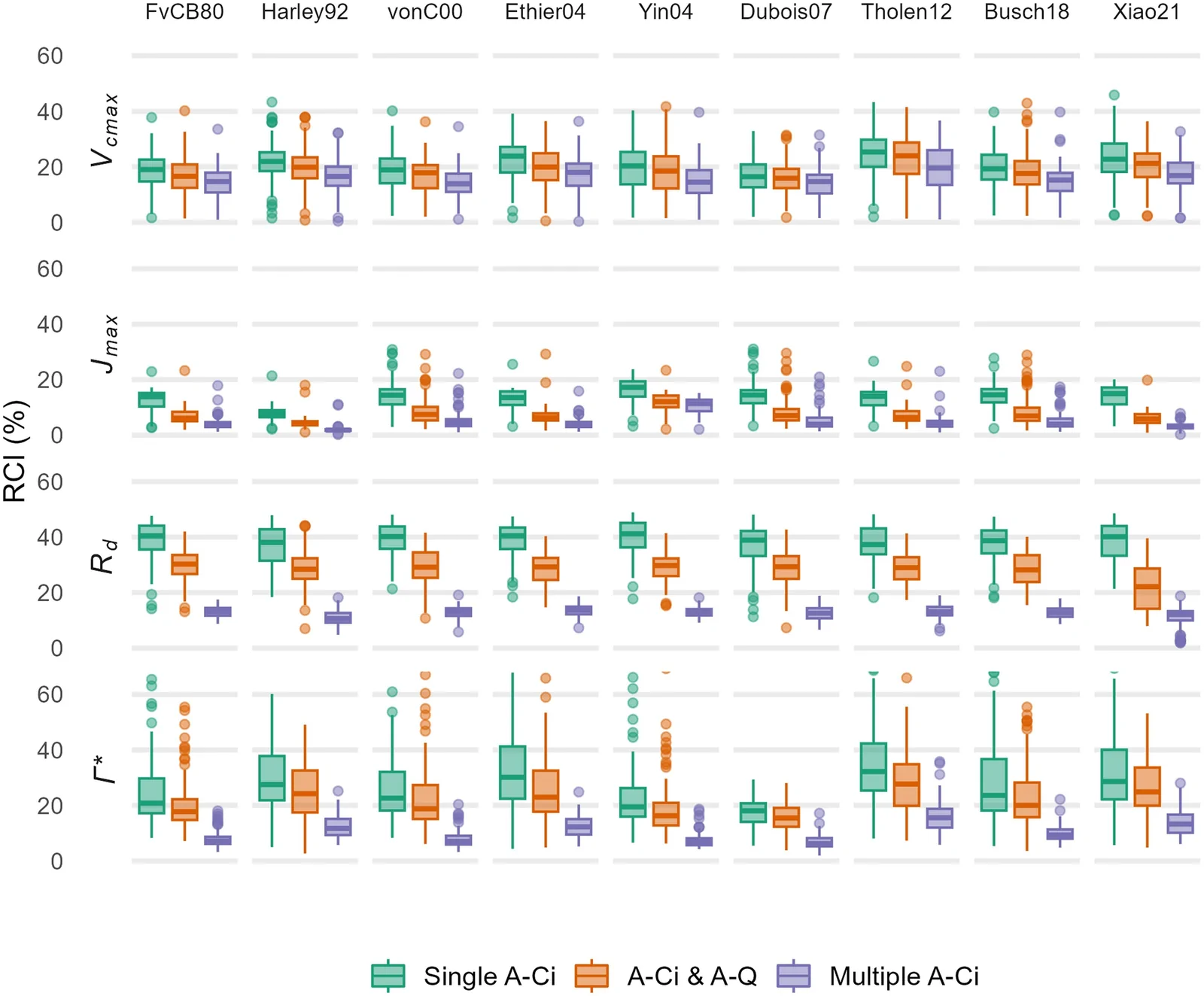
Relative widths of credible intervals for parameters across the nine FvCB models. Shown are the relative width of the 50% highest posterior density intervals obtained from the parameter posterior distributions generated by Bayesian inference of the four parameters: maximum rate of RuBP-saturated carboxylation, *V*_cmax_, maximum rate of ET through PSII, *J*_max_, leaf mitochondrial respiration, *R*_d_, and CO_2_ compensation point in the absence of dark respiration, Γ*. From left to right within each model group, the three boxplots represent the RCI of parameter estimated obtained using : (1) a single *A–C*_i_ curve simulated at saturating light (1800 µmol m^−2^ s^−1^ PPFD), (2) *A–C*_i_ curve at saturating light together with *A–Q* curve at 300 µmol CO_2_ mol^−1^, and (3) *A–C*_i_ curves simulated at multiple light levels.

The RCI widths for *K*_C_ and *K*_O_ were consistently large (>40%; Supplementary Fig. S2A) across all models and data types, reflecting their structural non-identifiability: different combinations of *K*_C_ and *K*_O_ can yield the same composite term *K*_CO_=*K*_C_ (1+O/*K*_O_) at a fixed oxygen level. To address this issue, we re-parameterized the models by treating *K*_CO_ as a parameter. This approach markedly reduced uncertainty for *K*_CO_, yielding narrower CIs (∼20%) across all data types compared with estimating *K*_C_ and *K*_O_ separately (Supplementary Fig. S3). This re-parameterization did not alter the uncertainty patterns of other parameters; therefore, *K*_CO_ was used as a parameter, replacing *K*_C_ and *K*_O_ in the subsequent analyses.

Among the parameters specific to individual model variants, *g*_m_ in the Harley92, Ethier04, and Xiao21 models showed narrower RCI widths when multi-light *A–C*_i_ curves were applied (Supplementary Fig. S2B). Similarly, in the Tholen12 model, the conductance through the chloroplast envelope and stroma, *g*_ch_, and the conductance through the chloroplast wall and the plasmalemma, *g*_wp_, showed slightly narrower RCI width when multi-light *A–C*_i_ curves were fitted. For parameters associated with the light reactions, we found significant improvements when multi-light *A–C*_i_ curves were included: the maximum ET efficiency of PSII, Φ_IImL_, in the Yin04 model, the maximum quantum efficiency of PSII under low light, Φ_IIL_, and the combined parameter of light absorption and partition to PSII, *s*, in the Xiao21 model.

In contrast, the TPU rate (*V*_TPU_) in the vonC00, Dubois07, and Busch18 models showed only a slight reduction in RCI widths under multi-light *A–C*_i_ fitting and exhibited greater variability across repetitions. Interestingly, the glycolate carbon export fraction, α_G_, in the vonC00 model showed higher uncertainty (RCI width >40%; Supplementary Fig. S2B) with no improvement from additional *A–C*_i_ curves considered, whereas in the Dubois07 model its uncertainty was substantially lower (RCI width ∼20%). Related parameters in the Busch18 model, including the maximum glycolate carbon fraction exported as glycine, max_αG_, and as serine, max_αS_, and the maximum rate of *de novo* nitrogen supply to the chloroplast, *N*_max_, showed moderate uncertainty and did not significantly benefit from multi-light *A–C*_i_ fitting.

While photosynthesis limited by Rubisco and RuBP regeneration represents the dominant operating regime across most simulated conditions, TPU limitation was not consistently present across the sampled parameter space. Among simulations using the vonC00, Dubois07, and Busch18 models, 30 out of the 100 sampled parameter sets produced *A–C*_i_ curves exhibiting TPU limitation under saturating light conditions, indicating that the sampling spanned a broad parameter space and captured diverse physiological regimes (Supplementary Fig. S4A). The uncertainty of *V*_TPU_ estimated from these curves with TPU limitation was lower across all three models than that obtained from curves without TPU limitation (Supplementary Fig. S4B). A similar, although weaker, reduction in uncertainty was observed for α_G_ in the vonC00 model and *N*_max_ in the Busch18 model when TPU limitation was expressed. In contrast, max_αG_ and max_αS_ in the Busch18 model showed no reduction under the constraint from TPU limitation. Notably, α_G_ in the Dubois07 model was the only parameter exhibiting increased uncertainty given TPU limitations, potentially reflecting differences in how *A*_n_ is formulated.

### Accuracy of parameter estimation across different models and response curves

The analyses above evaluated parameter uncertainty when all parameters of each model variant were estimated simultaneously. While this setting provides a comprehensive assessment of identifiability, it does not reflect the common practice in many photosynthesis-fitting tools. The existing tools estimate only a subset of parameters, while the remaining parameters are fixed to values obtained from other sources (e.g. the literature). To assess how this strategy affects estimation accuracy, we compared three parameterization scenarios that differed in how non-core parameters were treated.

In the first scenario, all model parameters were estimated simultaneously for each model and three synthetic data configurations: single *A–C*_i_, combined *A–C*_i_ and *A–Q*, and multi-light *A–C*_i_ curves. This corresponds to the setting used in the uncertainty analysis above. In the second scenario, only a core subset of parameters (i.e. *V*_cmax_, *J*_max_, *R*_d_, and Γ*) was estimated, while all remaining non-core parameters were fixed to the median of their sampled distributions (Supplementary Fig. S1A). This represents a practical situation in which fixed literature- or population-level parameter values are assumed to be known *a priori* and are therefore used for parameters that are not estimated. In the third scenario, the same core parameters were estimated, but the non-core parameters were fixed to their true sampled values, representing a best-case situation in which these parameters are known without error.

Comparing these three scenarios allowed us to determine how different levels of prior knowledge about non-core parameters affect the estimation accuracy of the core parameters. To quantify the comparison, we assessed estimation accuracy using the MAP estimates obtained from Bayesian inference. For each synthetic data configuration and parameterization scenario, accuracy was calculated as Pearson’s correlation coefficient, *r*, between the MAP estimates and the corresponding true parameter values represented by the 100 sampled parameter sets.

When all parameters were estimated simultaneously, *V*_cmax_, *J*_max_, and Γ* already showed high accuracy when only a single *A–C*_i_ curve was used for parameter estimation (mean *r* across models >0.6; Fig. 3). In contrast, *R*_d_ was weakly constrained by single *A–C*_i_ data, but its estimation accuracy increased markedly when additional dimensions were introduced. Specifically, combining *A–C*_i_ and *A–Q* data and, more strongly, using *A–C*_i_ curves at multiple PPFDs substantially improved *R*_d_ estimation, with mean correlations increasing from 0.30 (single *A–C*_i_ ) to 0.93 (multi-light *A–C*_i_ ).

**Fig. 3.**
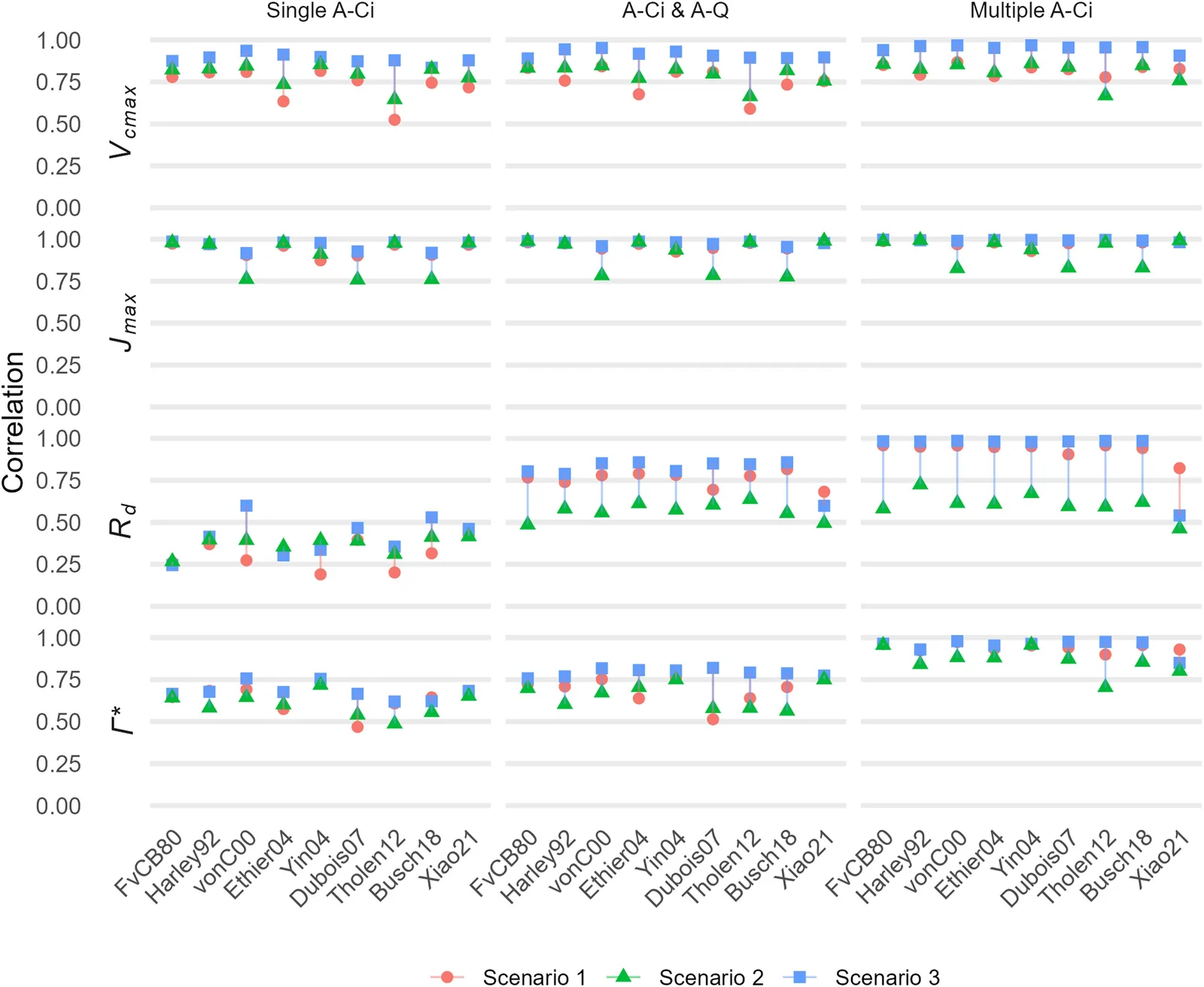
Parameter estimation accuracy across models using three estimation strategies. Pearson’s correlation coefficient between maximum *a posteriori* (MAP) estimates and the corresponding true parameter values from the 100 sampled parameter sets for the four core parameters are shown. In the first scenario, all model parameters are simultaneously estimated (circle). In the second scenario, only the core parameters are estimated, with remaining parameters fixed to their median values (triangle, Supplementary Fig. S1). The third scenario differs from the second by fixing the remaining parameters to their true sampled values and therefore represents an upper bound on achievable estimation accuracy for the core parameters (square).

When only core parameters were estimated and all non-core parameters were fixed to their median values, estimation accuracy declined relative to the fully estimated case for several parameters. This reduction was most pronounced for *R*_d_, for which correlations decreased across all models and data types. Notably, increasing data with multiple light levels did not compensate for the observed loss, indicating strong confounding between *R*_d_ and fixed non-core parameters. In contrast, fixing non-core parameters improved the estimation of *V*_cmax_ across all data types, suggesting reduced parameter trade-offs. For *J*_max_, the correlations obtained under the second scenario were generally comparable with those from the first scenario, with a notable decrease for the vonC00, Dubois07, and Busch18 models.

As expected, fixing non-core parameters to their true values yielded the highest estimation accuracy, providing an upper bound on achievable performance. For *J*_max_, estimation accuracy obtained from a single *A–C*_i_ curve under the first two strategies already approached this upper bound and did not improve further with additional data. In contrast, the upper bound for *R*_d_ and Γ* depend strongly on the type of response curves used, with multi-light *A–C*_i_ sampling providing the highest limit. When only a single *A–C*_i_ curve was used for fitting, differences in the estimation accuracy among model formulations were more pronounced; however, these differences were progressively reduced as additional response data were incorporated.

An advantage of estimating all parameters simultaneously is that it allows evaluation not only of core photosynthetic parameters but also of additional parameters, including both those shared across models and those specific to individual FvCB formulations. Although *K*_CO_ exhibited lower uncertainty than *K*_C_ and *K*_O_, its MAP estimates still showed weak correlations with the true values (mean *r* = 0.20, 0.23, and 0.29 for the three data types; Supplementary Fig. S5), with the strongest performance observed for the Harley92 model when multi-light *A–C*_i_ curves were applied.

Among the model-specific parameters, *g*_m_ showed correlation increasing from 0.44 (single *A–C*_i_) to up to 0.71 (multi-light *A–C*_i_, Supplementary Fig. S5). Moderate correlations were observed for Φ_IIm_ and *h* in the Yin04 model and for Φ_IILL_ in the Xiao21 model when multi-light *A–C*_i_ curves were fitted (*r*∼0.5). Further, we found Pearson’s correlations of 0.82 and 0.65 using multi-light *A–C*_i_ curves for *g*_ch_ and *g*_wp_ in the Tholen12 model, respectively. The remaining parameters did not show marked improvement by adding more data points. Some parameters, such as *N*_max_ in the Busch18 model and Φ_IILL_ in the Xiao21 model, showed narrower posterior distributions when multi-light *A–C*_i_ curves were used, indicating improved practical identifiability. However, their MAP estimates still showed weak correlation with the true values. Complementary findings based on the relative root-mean-square error (RMSE) of estimated parameters were consistent with those based on Pearson correlations (Supplementary Fig. S6).

In terms of the curve fitting accuracy, the difference between datasets used in model fitting was consistent across most models (Supplementary Fig. S7). We found that when *A–Q* or multi-light *A–C*_i_ curves were included, both the median RMSE and interquartile ranges were reduced, suggesting a more accurate and reliable fit.

### Estimation consistency and model selection using real gas exchange data

In the previous analyses, estimation uncertainty and accuracy were examined using synthetic data configurations generated from randomly sampled parameter sets. Building on the findings, we next assessed the extent of consistency between the parameter estimates across models when using data from real-world gas exchange measurements. Simultaneous estimation of parameters with Bayesian inference was applied to *A–C*_i_ and *A–Q* response curves measured from 85 barley genotypes (Breil-Aubert *et al.*, 2025). Each genotype was grown in two plots, and three plants were measured per plot, resulting into a total of 354 response curve measurements across 156 plots. Separate plant-, plot-, and genotype-specific datasets were assembled (see Supplementary Fig. S8 for illustration of the first 15 genotypes) and analyzed independently to evaluate the robustness of estimated parameter values using data at different data levels.

To evaluate the robustness of the estimated parameters, we averaged the plant-level parameter estimates within each plot or genotype to obtain plot- and genotype-level values, and then compared these aggregated estimates with the parameter estimated directly at those data levels (see the Materials and methods). Parameters that previously exhibited relatively narrow RCI widths and moderate to high estimation accuracy using synthetic data, namely *V*_cmax_, *J*_max_, *R*_d,_, Γ*, θ_J_, α_J_, *g*_m_, and *g*_ch_, also showed higher robustness between different data levels (Supplementary Fig. S9). In contrast, the estimates for parameters with larger RCI widths, including the fraction of the glycolate carbon exported from the chloroplast, α_G_, the fraction of pseudocyclic ET, *f*_pseudo_, and the maximum glycolate carbon fraction exported as glycine and serine, max_αG_ and max_αS_, lacked consistency between data levels. The similarity between models were quantified by calculating the average Euclidean distance over genotypes between the estimates of shared parameter and the associated CV (Fig. 4, see the Materials and methods).

**Fig. 4.**
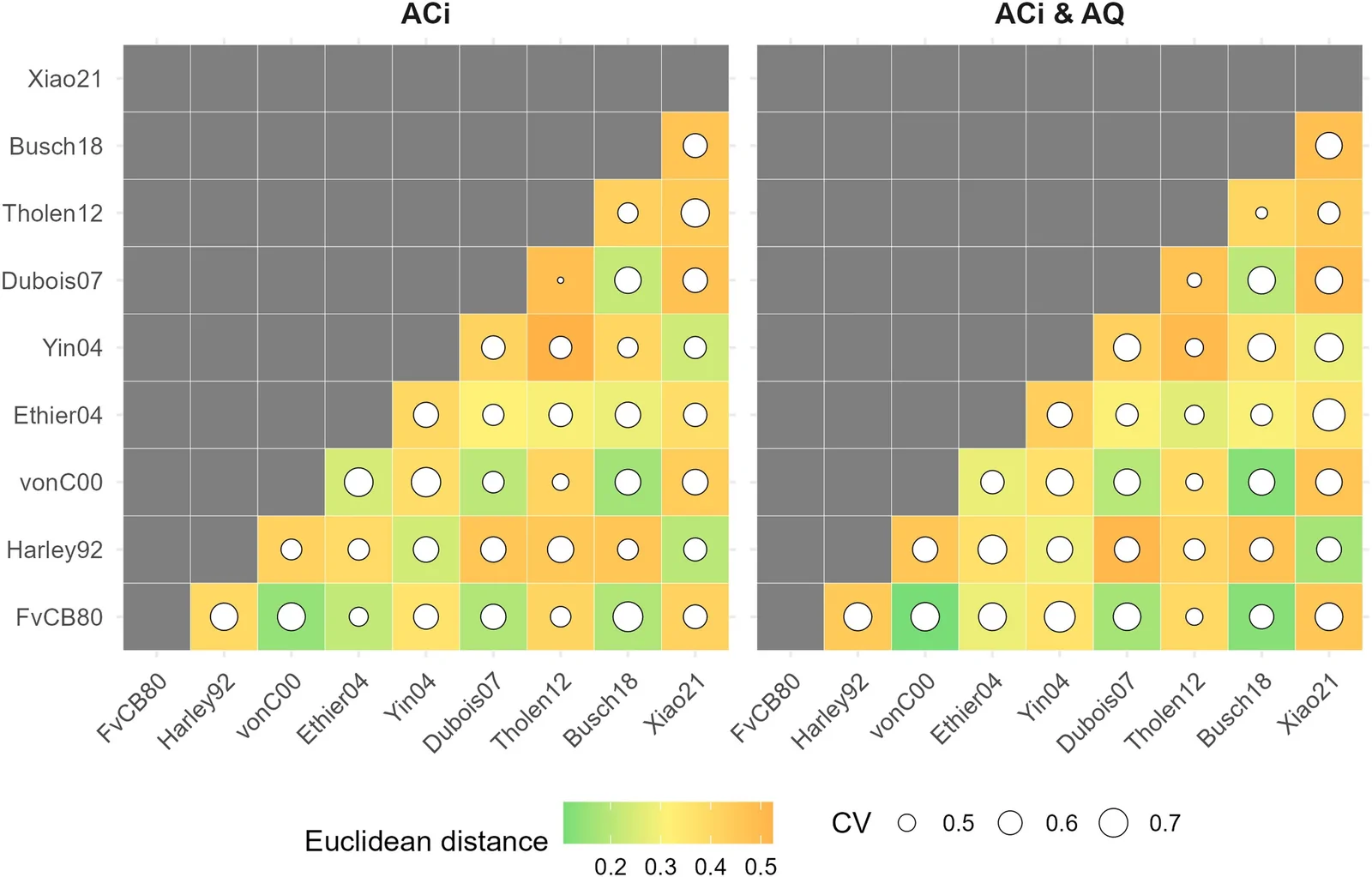
Pairwise comparison between models using genotype-level *A–C*_i_ curves alone and combined *A–C*_i_ and *A–Q* curves. Mean Euclidean distance of parameter estimates between models. Two datasets were used for estimation. The radius of the circles represents the coefficient of variation of Euclidean distance across genotypes.

We found that the vonC00, Busch18, and Dubois07 models were the closest to the *F*vCB80 model when *A–C*_i_ curves we used as data input (Supplementary Fig. S10A). Another group of models comprised the Harley92, Yin04, and Xiao21 models, while a third group included the Tholen12 and Ethier04 models (Supplementary Fig. S10B). The CV of the Euclidean distances across genotypes (represented by circle radius in Fig. 4) indicated that the variability of parameter similarity across genotypes was slightly higher when *A–C*_i_ and *A–Q* curves were combined. For example, the parameter 74 showed a small average distance, with smaller variation in the distance across genotypes, confirming their similarity in parameter estimates.

In terms of curve fitting accuracy, when only *A–C*_i_ curves were used for model fitting, the Busch18 model yielded the lowest median RMSE at the individual plant level, while the vonC00 model achieved the lowest RMSE at both plot and genotype level (Supplementary Fig. S11). The same pattern was observed when *A–C*_i_ and *A–Q* curves were jointly fitted.

To identify the best-performing model, we calculated the AIC based on the maximized log-likelihood and the number of parameters (see the Materials and methods). When fitting to single *A–C*_i_ curves, the vonC00 model yielded the best (i.e. smallest) AIC score at the plant level, while the FvCB80 model exhibited the best AIC score at the plot and genotype level (Supplementary Fig. S12). For the combined *A–C*_i_ and *A–Q* data, the FvCB80 model showed the best overall performance across all levels.

## Discussion

Several FvCB-type model variants have been developed to extend the original formulation and represent additional biochemical or biophysical processes, including TPU limitation, alternative ET pathways, and mesophyll CO_2_ diffusion (Bernacchi *et al.*, 2013; Von Caemmerer, 2013; Yin *et al.*, 2021; Lochocki and McGrath, 2025). Parallel to these model developments, various tools were implemented to estimate FvCB parameters, most commonly using non-linear least square approaches implemented in Excel (Sharkey *et al.*, 2007; Bellasio *et al.*, 2016; Sharkey, 2016) and R packages (Duursma, 2015; Gregory *et al.*, 2021; Stinziano *et al.*, 2021; Lochocki *et al.*, 2025). Other optimization algorithms have been explored, including genetic algorithms (Su *et al.*, 2009), hybrid search methods (Gu *et al.*, 2010), gradient-based optimizers (e.g. ADAM) (Lei *et al.*, 2025), and Bayesian inference frameworks (Patrick *et al.*, 2009; Xiao *et al.*, 2021).

Despite these advances and the recent release of the R and Python packages, including: Plantecophys (Duursma, 2015), Photosynthesis (Stinziano *et al.*, 2021), PhotoGEA (Lochocki *et al.*, 2025), and PhoTorch (Lei *et al.*, 2025), that automate parameter estimation from gas exchange data, several challenges remain. First, most packages are implemented using a single FvCB variant, limiting model comparability, with the exception of PhotoGEA, which includes both the vonC00 and Busch18 models, as well as the Variable J method for *g*_m_ calculation. Second, many parameters are fixed to default values, which may not be valid across species or conditions. Third, achieving a low fitting error does not necessarily ensure accurate parameter estimation; therefore, the identifiability and uncertainty of parameters given the available data must be systematically assessed.

Previous studies have addressed important aspects of FvCB model ambiguity and parameter estimation reliability. Lochocki and McGrath (2025) examined ambiguity arising from mathematical discrepancies among FvCB formulations and identified specific sources of estimation error associated with alternative formulations and the omission of TPU limitation. Xiao *et al.* (2021) used a Bayesian framework to evaluate the reliability of FvCB parameter estimation and showed that the choice of fixed versus estimated parameters can strongly influence posterior uncertainty and estimation accuracy. Together, these studies demonstrate that reliable parameter estimation depends on the interaction between model formulation, data availability, and parameter treatment decisions. However, these factors have rarely been evaluated together across a broad set of FvCB variants under a common inference framework.

To address these limitations, we compiled nine FvCB model variants that capture the four key extensions above and used Bayesian inference to explore three critical questions guiding practical parameter estimation. (i) Which parameters can be estimated accurately? (ii) Which model provides the best fit? (iii) Which types of response curves are required for reliable estimation?

Each FvCB model is defined as a set of equations describing *A*_n_ as a function of environmental conditions (*C*_i_ and PPFD) and a parameter vector (Table 1). We sampled 100 sets of parameter values and treated these as true values to generate three types of synthetic *A*_n_ data for each model: a single *A–C*_i_ curve at saturating light, combined *A–C*_i_ and *A–Q* curves, and *A–C*_i_ curves at multiple PPFD levels. By applying Bayesian inference to estimate parameters for each model from each synthetic data configuration, we assessed the uncertainty and accuracy of parameter estimation (Fig. 2).

We found that the relative widths of the credible intervals, expressed as a percentage of the sampling range, were small for *V*_cmax_, *J*_max_, and Γ*, even when fitting a single *A–C*_i_ curve, indicating low uncertainty and high precision. These parameters also showed the strongest prediction accuracy, with Pearson’s correlations between true and estimated values exceeding 0.6. In contrast, precise and accurate estimation of *R*_d_, θ_J_, and α_J_ required *A–C*_i_ curves generated under multiple PPFD levels. This is consistent with the rationale of dedicated approaches for estimating respiration in the light, such as Kok- and Laisk-type measurements, which use low-light or low-*C*_i_ measurements at multiple irradiances to estimate *R*_d_ more directly from the intercept or intersection (Kok, 1948; Laisk, 1977). A standard *A–C*_i_ curve measured under a single saturating irradiance contains limited information about *R*_d_, because changes in *R*_d_ can be partly compensated by changes in carboxylation-related parameters during model fitting. By contrast, varying irradiance changes the balance between light-dependent ET and CO_2_-dependent carboxylation, thereby providing additional information to constrain parameters related to respiration and light response.

Neither *K*_C_, *K*_O_, nor their composite term *K*_CO_ could be reliably estimated when only a single O_2_ concentration (210 mmol mol^−1^) was available. Model-specific parameters related to mesophyll conductance (e.g. *g*_m_, *g*_wp_, and *g*_ch_) and to ET efficiency (e.g. Φ_IIm_, Φ_IILL_, and *s*) exhibited markedly improved identifiability when multi-light *A–C*_i_ curves were applied. For the TPU-related parameters, *V*_TPU_ and α_G_, the vonC00 model showed modest improvement when combining *A–C*_i_ and *A–Q* curves. Moreover, the maximum rate of *de novo* nitrogen supply to the chloroplast, *N*_max_, from the Busch18 model showed larger improvement with multi-light *A–C*_i_ curves (Supplementary Fig. S4). These findings demonstrate that the parameter posterior distribution strongly depends on the type of response curves used for model fitting. They also point out that the parameter posterior distribution must be carefully investigated, particularly if the non-core parameters are of interest.

Across the latest parameter estimation tools, such as Plantecophys (Duursma, 2015), Photosynthesis (Stinziano *et al.*, 2021), PhotoGEA (Lochocki *et al.*, 2025), and PhoTorch (Lei *et al.*, 2025), the default estimated parameters are typically *V*_cmax_, *J*_max_, *R*_d_, and Γ*, with all other parameters fixed or optionally optimized (see Supplementary Table S1 for differences in variables and default values between tools). A critical methodological decision in photosynthesis model calibration is whether to estimate only a default subset of parameters while fixing the remaining ones, or to treat all parameters as free variables during optimization. Using synthetic data, we found that simultaneous estimation of all parameters generally improved estimation accuracy relative to fitting only a subset of core parameters, with the remaining parameters fixed to the median sampled value (Supplementary Fig. S5). Among the core parameters, *R*_d_ was particularly sensitive to the values assigned to non-core parameters. Notably, we observed a small difference between estimating all parameters and the idealized scenario, where only core parameters are estimated while non-core parameters are fixed to their true values. This finding indicated that joint estimation of all parameters can improve estimation accuracy even without perfect prior knowledge of non-core parameters. In addition, differences in estimation accuracy among FvCB models were most pronounced when fitting a single *A–C*_i_ curve and reduced as additional response data were incorporated. This highlights that the choice of phenotyping strategy, particularly the inclusion of complementary measurements that expand *A–C*_i_ curves across environmental space, can mitigate model structure-dependent differences and improve parameter estimation.

Since different tools implement distinct FvCB variants, the choice of software implicitly constrains the model structure and estimated values. Using real *A–C*_i_ and *A–Q* response curves measured across 85 barley genotypes, we further evaluated the robustness of the estimation using the three levels of data organization (Supplementary Fig. S8). For each genotype, up to six individual plant measurements were used for estimation (genotype-level estimates); for each plot, up to three plant-level measurements were included (plot-level estimates); while a single measurement resulted in plant-level estimates. The estimation of parameters across different data levels was used to assess the reliability of the estimation. Parameters that showed high consistency between plant- and plot-level estimates also exhibited higher estimation accuracy in the synthetic data analysis. This can be explained by the fact that plant- and plot-level response curves originated from the same genotype, sharing more similarity; therefore, estimates of more identifiable parameters also showed higher consistency between these levels. In contrast, for non-identifiable parameters, those that cannot be uniquely determined from the available data, the mean values estimated at the plant level often differed from those at the plot level, reflecting the instability of their estimation across data levels.

As expected, fitting accuracy decreased from plant- to genotype-level analyses due to the increasing data heterogeneity and the greater difficulty of identifying a single parameter set that simultaneously reproduces all measurements within these broader groups (Supplementary Fig. S9). Combining *A–C*_i_ and *A–Q* data improved the consistency of parameter estimates across different data levels compared with fitting only *A–C*_i_ curves, although the overall fitting performance was reduced due to the increased complexity of the data. Based on the AIC scores, the Harley92, vonC00, Tholen12, and Busch18 models exhibited the best performance across different data levels and datasets, indicating that model performance strongly depends on the type of data used for estimation (Supplementary Fig. S12).

Unlike existing studies, that primarily focused on implementing or comparing specific estimation algorithms (Dubois *et al.*, 2007; Sharkey *et al.*, 2007; Miao *et al.*, 2009; Su *et al.*, 2009; Gu *et al.*, 2010; Yin *et al.*, 2011; Sun *et al.*, 2014; Duursma, 2015; Sharkey, 2016; Gregory *et al.*, 2021; Stinziano *et al.*, 2021; Lei *et al.*, 2025; Lochocki *et al.*, 2025), our work aims to establish a comprehensive workflow for the entire parameter estimation process, considering the evaluation of credible intervals, types of fitted response curves, and level of estimation (plant, plot, or genotype level), and model selection. This broader framework serves as a guideline for three key decision points: (i) the type and combination of response curves required to obtain reliable estimates; (ii) the treatment of parameters as fixed or variable; and (iii) the choice of FvCB variant. Together, these factors jointly determine the accuracy of estimation and model consistency across biological and environmental levels.

Although hierarchical Bayesian approaches have previously been applied to FvCB parameterization (Patrick *et al.*, 2009; Han *et al.*, 2020), future research could further advance this direction by systematically identifying which parameters vary among genotypes or individual plants through integrated sensitivity analyses. In parallel, future software development should incorporate automated evaluation of parameter uncertainty based on the available data and explicitly indicate which parameters are reliably estimable and which are not. Additional extensions should include temperature response formulations for specific parameters and offer modular components for different biochemical processes within photosynthesis, enabling users to specify whether to incorporate alternative electron transport, as well as detailed formulations for mesophyll conductance and TPU limitation.

## Supplementary Material

erag340_Supplementary_Data

## Data Availability

All code and synthetic data to ensure reproducibility of the results can be accessed at: https://github.com/Rudan-X/FvCBestimation.

## Supplementary data

The following supplementary data are available at *JXB* online.

Table S1. Summary of six tools for estimation of photosynthetic parameters by fitting to gas exchange data and chlorophyll fluorescence.

Fig. S1. Sampled parameter distributions used to generate synthetic *A–C*_i_ curves across the nine FvCB models.

Fig. S3. Relative widths of credible intervals for the parameters across the nine FvCB models when *K*_CO_ was estimated.

Fig. S4. Occurrence of TPU limitation in simulated *A–C*_i_ curves and its impact on parameter uncertainty.

Fig. S6. Relative RMSE between MAP estimates and true parameter value across 100 random samples for all parameters.

Fig. S7. Overall RMSE of curve fitting across FvCB models and response curves.

Fig. S8. Distribution of measured *A–C*_i_ and *A–Q* response curves for the first 15 genotypes.

Fig. S9. Consistency of parameter estimates across biological aggregation levels.

Fig. S10. Dendrogram of nine FvCB models based on Euclidean distance.

Fig. S11. Summary of curve fitting accuracy across FvCB models using barley gas exchange data.

Fig. S12. Selection of FvCB models using barley gas exchange data based on AIC score.
