# Supplementary material for: Parameterizing steady-state C3 photosynthesis models using photosynthesis response curves: which models to use and which parameter estimates to trust?
Source: J Exp Bot. 2026 Jul 17;77(19):6609–23. doi: 10.1093/jxb/erag340 (PMC13672984; doi:10.1093/jxb/erag340)
Supplement: Supplementary file 1 [file erag340_supplementary_data.pdf]

Supplementary Information

Parameterizing steady-state C_3 photosynthesis models using photosynthesis response curves: Which models to use and which parameter estimates to trust?

Rudan Xu[1,2], John N. Ferguson[3], Salma Tariq[1], Patience B. Iliya[1,2], Anika Küken[1], Johannes Kromdijk[4], Zoran Nikoloski[1,2,*]

[1]Bioinformatics Department, Institute of Biochemistry and Biology, University of Potsdam, Potsdam, Germany

[2]Systems Biology and Mathematical Modelling Group, Max Planck Institute of Molecular Plant Physiology, Potsdam, Germany

[3]School of Life Sciences, University of Essex, Colchester, UK

[4]Department of Plant Sciences, University of Cambridge, Cambridge, UK

[*]Corresponding author

Contact: nikoloski@mpimp-golm.mpg.de

Supplementary Table S1. Summary of six tools for estimation of photosynthetic parameters by fitting to gas exchange data and chlorophyll fluorescence. The table illustrates the differences among the steady-state photosynthesis model fitted to the gas exchange data, the types of input data handles by each tool, and the classification of model parameters depending on whether they are always fitted (Fit), optionally fitted (Optional fit), user-defined with argument (User-defined), or fixed within the inbuilt function (with the corresponding fixed value indicated).

	Plantecophys (2015)	*PCEcalculator* (2016)	*msuRACiFit* (2021)	*Photosynthesis* (2021)	*PhotoGEA* (2025)	*PhoTorch* (2025)
**Fitting A-Ci curves**						
Model	Collatz et al. (1991)	von Caemmerer (2000)	Busch et al. (2017)	von Caemmerer (2000)	Busch, (2020)	Ethier and Livingston (2004)
Data	A-Ci	A-Ci	A-Ci (& CF)	A-Ci (& CF)	A-Ci (& CF)	A-Ci
V_{cmax}	Fit	Fit	Fit	Fit	Fit	Fit
R_d	Fit	Fit	Fit	Fit	User input/ Fit	1.5
Γ^*	42.75/ User input	3.74 (Pa)	3.74 (Pa)	42.75/ User input	40/ Fit	42.75
K_C	404.9	27.24 (Pa)	27.24 (Pa)	User input	400/ Optional fit	404.9
K_O	278.4	16.58 (kPa)	16.58 (kPa)	User input	275/ Optional fit	278.4
$K_{CO,210}$	710.3	62.19 (Pa)	62.19 (Pa)	718.4/ User input	705.5	710.3
J_{max}	Fit	-	-	Fit	-	Fit
J	-	Fit	Fit	-	Fit	-
θ_J	0.85	-	-	0.85/ User input	-	0.7
α_J	0.24	-	-	0.24/ User input	-	0.5
V_{TPU}	1000/ Optional fit	Fit	Fit	Optional fit/ 1000	Fit	Fit
α_G	0/ Optional fit	-	Fit	Optional fit/ 0	0/ Optional fit	0
g_m	User input	Fit	Fit	User input/ Variable J	User input/ Optional fit/ Variable J	10/ Input
**Fitting A-Q curves**						
Model	-	Buckley and Diaz-Espejo (2015)	-	Marshall and Biscoe (1980)	-	-
Data	-	J-Q	-	A-Q	-	-
J_{max}	-	Fit	-	Fit	-	-
J	-	Calculated from A-Q	-	-	-	-
θ_J	-	Fit	-	Fit	-	-
α_J	-	Fit	-	Fit	-	-

R_d	-	User input	-	-	-	-
g_m		User input		-		

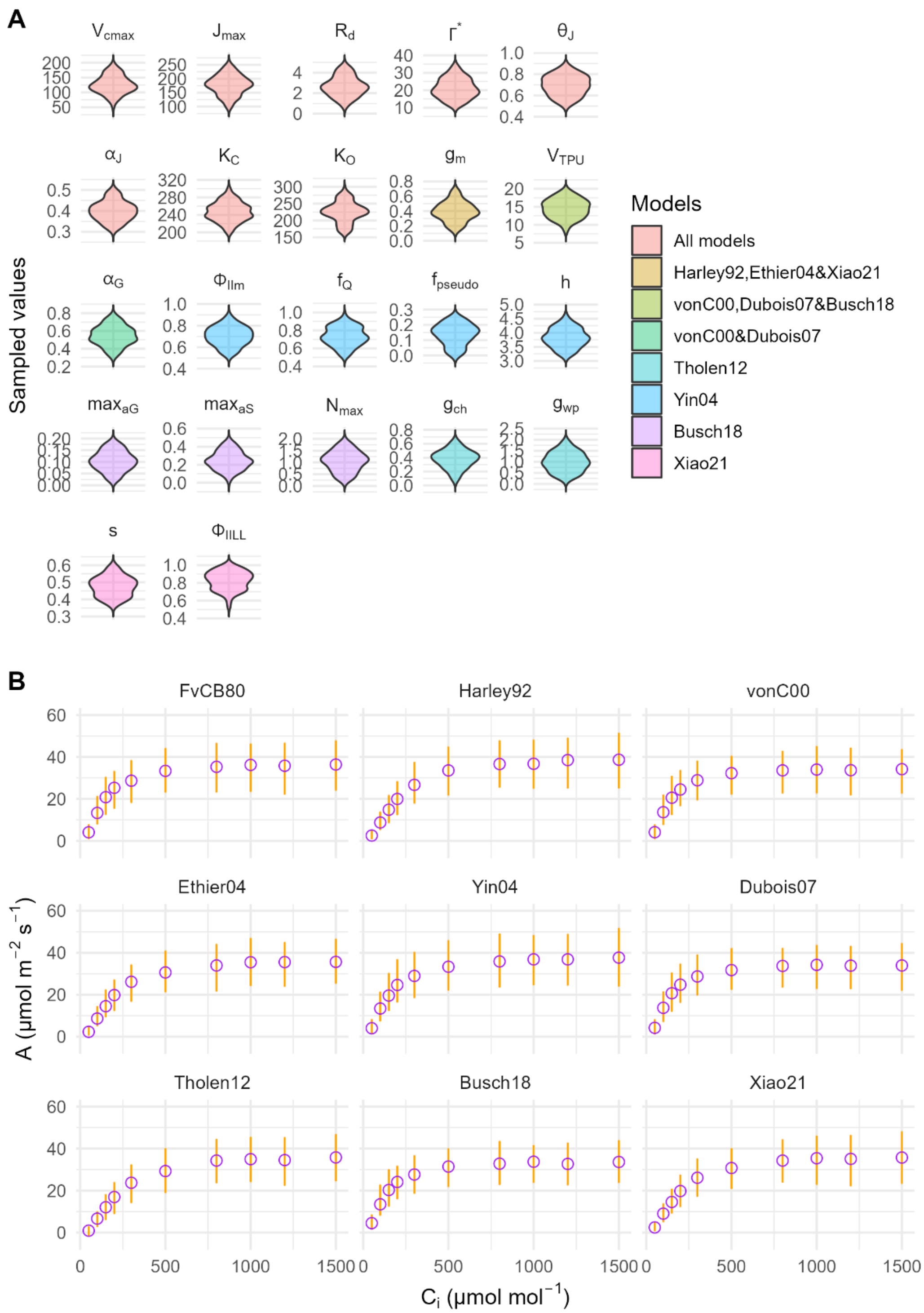

Supplementary Fig. S1. Sampled parameters distributions used to generate synthetic A-Ci curves across the nine FvCB models. (a) Violin plots show the distributions of the 100 sampled parameter sets. These sampled parameters were used to generate the synthetic gas exchange data. Colors indicate the model(s) in which each parameter is included. (b) Simulated A-Ci at 1800 μmol m^{-2} s^{-1} PPFD using the sampled parameter sets. The vertical orange lines show the 95% CI while the purple circles represent the median simulated A_n.

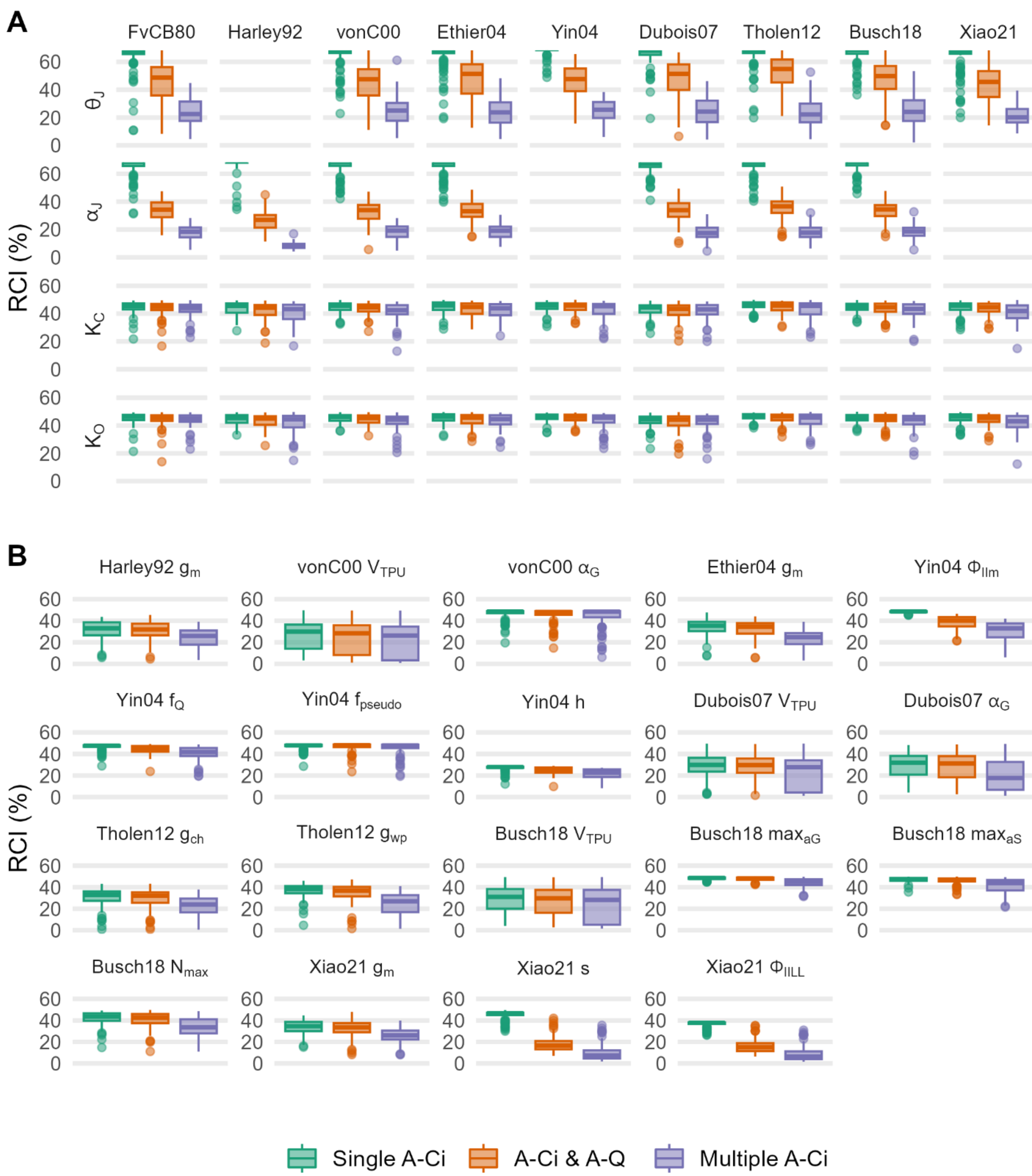

Supplementary Fig. S2. Relative widths of credible intervals for the remaining parameters across the nine FvCB models. The boxplots show the relative width of the 50% highest posterior density intervals obtained from the parameter posterior distributions for (a) shared parameters across models and (b) model-specific parameters. Colors represent three types of data used for parameter estimation: A-Ci curve simulated at saturating light (1800 µmol m^{-2} s^{-1} PPFD, green), A-Ci curve at saturating light and A-Q curve at 300 µmol CO_2 mol^{-1} (orange), and A-Ci curves simulated at different light levels (purple).

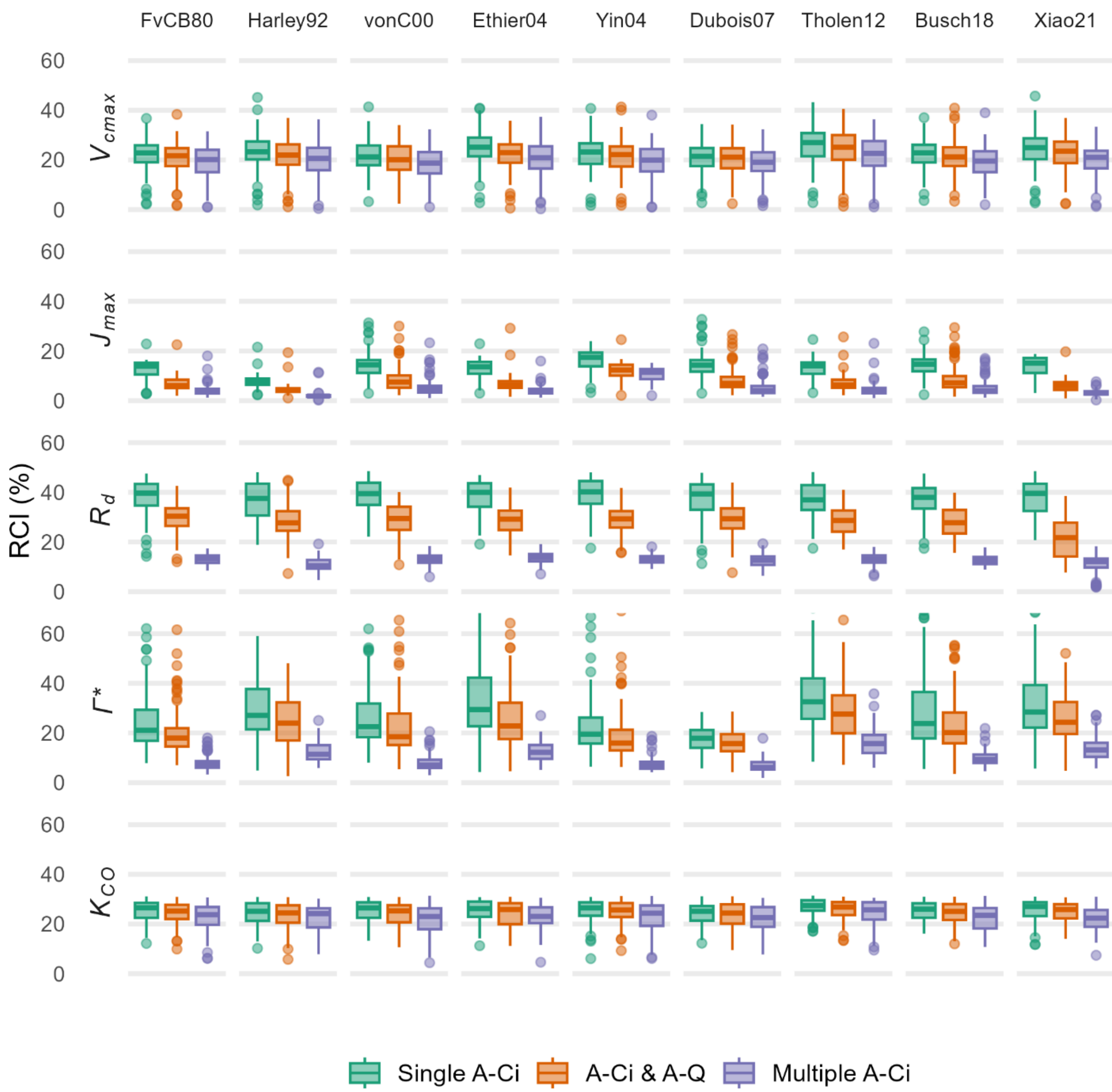

Supplementary Fig. S3. Relative widths of credible intervals for the parameters across the nine FvCB models when K_{CO} was estimated. The boxplots show the relative width of the 50% highest posterior density intervals obtained from the parameter posterior resulting from Bayesian Inference for five parameters: V_{cmax}, J_{max}, R_d, Γ^* and the composite term K_{CO}. Colors represent three types of data used for parameter estimation: A-Ci curve simulated at saturating light (1800 μmol m^{-2} s^{-1} PPFD, green), A-Ci curve at saturating light and A-Q curve at 300 μmol CO_2 mol^{-1} (orange), and A-Ci curves simulated at different light levels (purple).

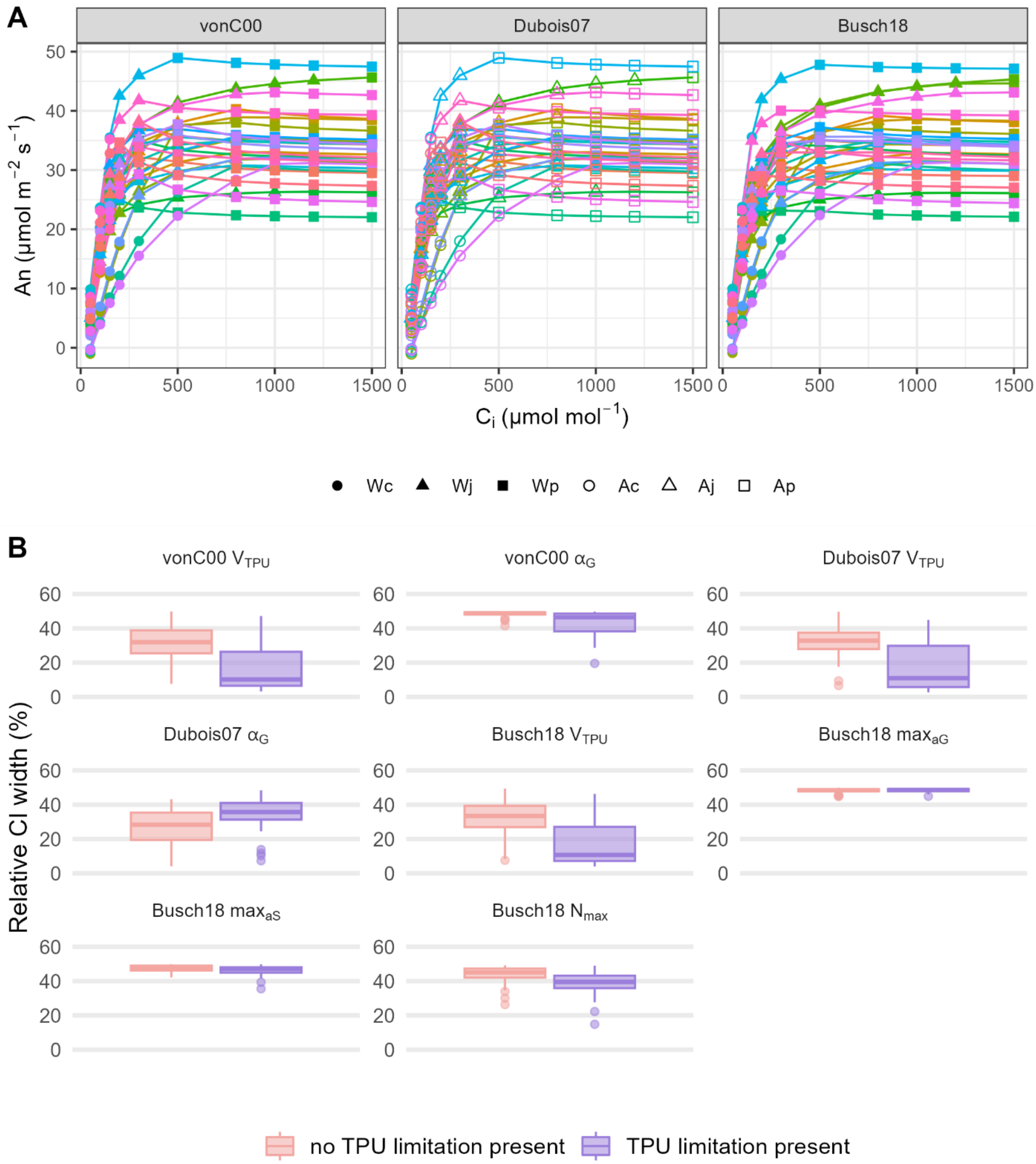

Supplementary Fig. S4. Occurrence of TPU limitation in simulated A-Ci curves and its impact on parameter uncertainty. (a) Simulated A-Ci curves at saturated light condition generated using three FvCB model variants that explicitly account for TPU limitation (*vonCaemmerer00, Dubois07, and Busch18*). Shown are the 30 noise-free simulated A–Ci curves (out of 100 total simulations per model) that exhibited TPU limitation, with the limiting process indicated by point symbols. (b) Relative CI widths of TPU-related parameters estimated from the simulated curves, comparing simulations without TPU limitation (70 cases; pink) and with TPU limitation (30 cases; purple).

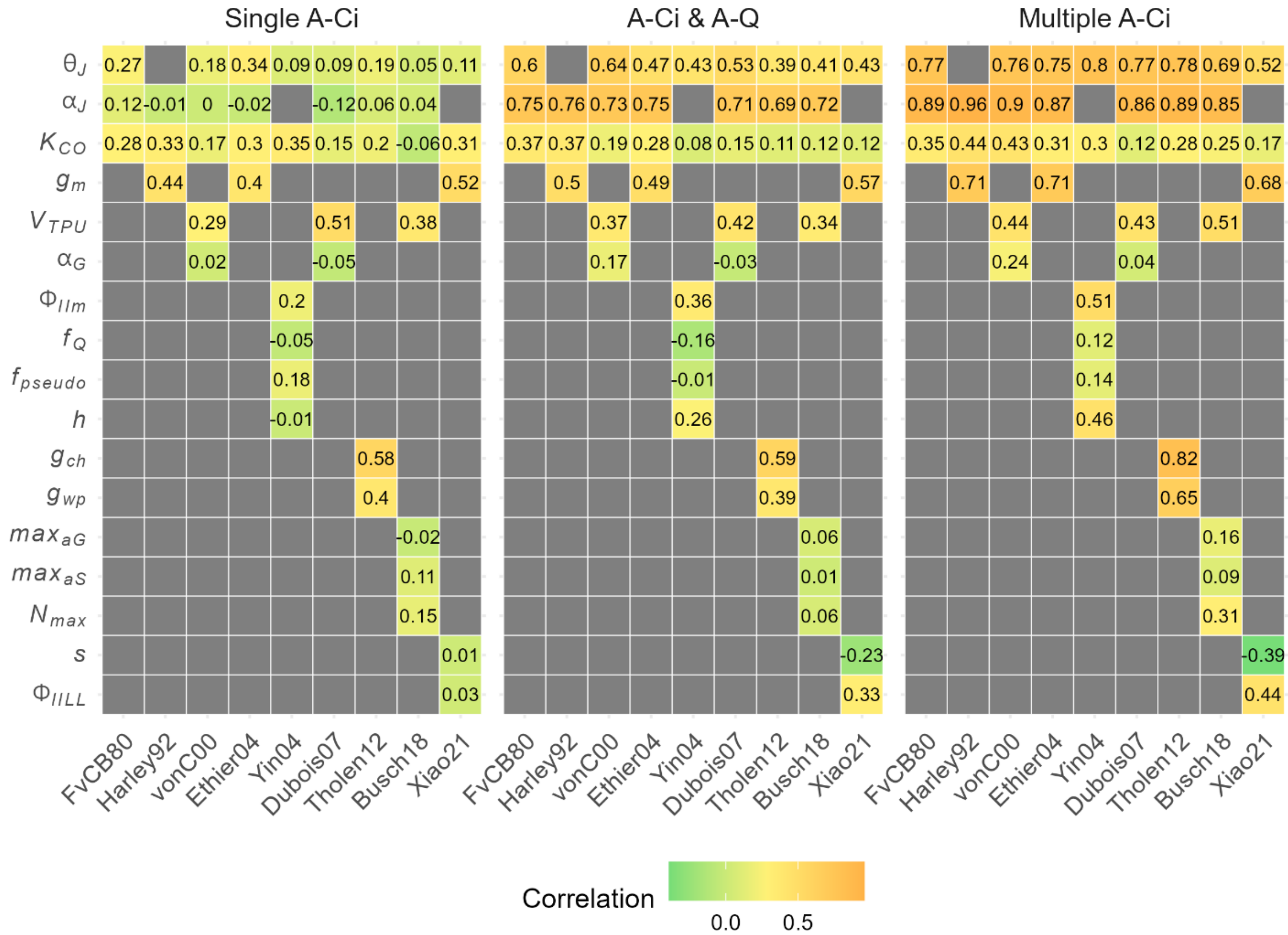

Supplementary Fig. S5. Accuracy of non-core parameter estimates under simultaneous estimation of all model parameters. The heatmap displays the Pearson correlation values between the MAP estimates and the corresponding true parameter values across the 100 sampled parameter sets. Results are shown for the three data types used for parameter estimation: (i) A-Ci curve simulated at saturating light (1800 µmol m^{-2} s^{-1} PPFD), (ii) A-Ci curve at saturating light and A-Q curve at 300 µmol CO_2 mol^{-1}, (iii) A-Ci curves simulated at different light levels. Gray cells indicate parameters not included in a given model.

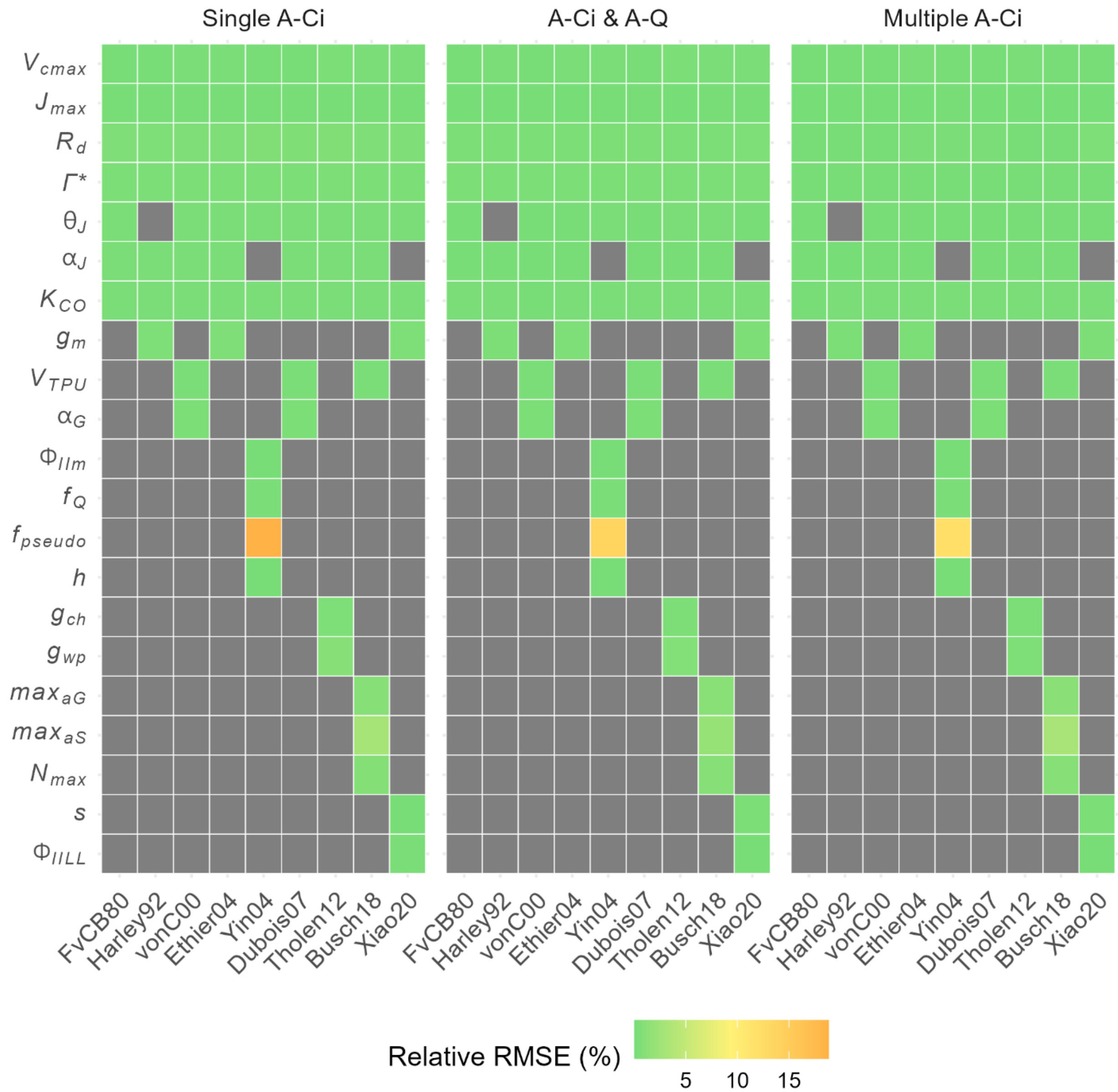

Supplementary Fig. S6. Relative RMSE between MAP estimates and true parameter value across 100 random samples for all parameters. Heatmaps depict the relative RMSE for parameters estimated using Bayesian inference across nine FvCB models under three types of response curves: A-Ci curve simulated at saturating light, A-Ci curve at saturating light, and A-Q curve at 300 µmol CO_2 mol^{-1}, and A-Ci curves simulated at different light levels. Parameters not included in a model are shown in gray.

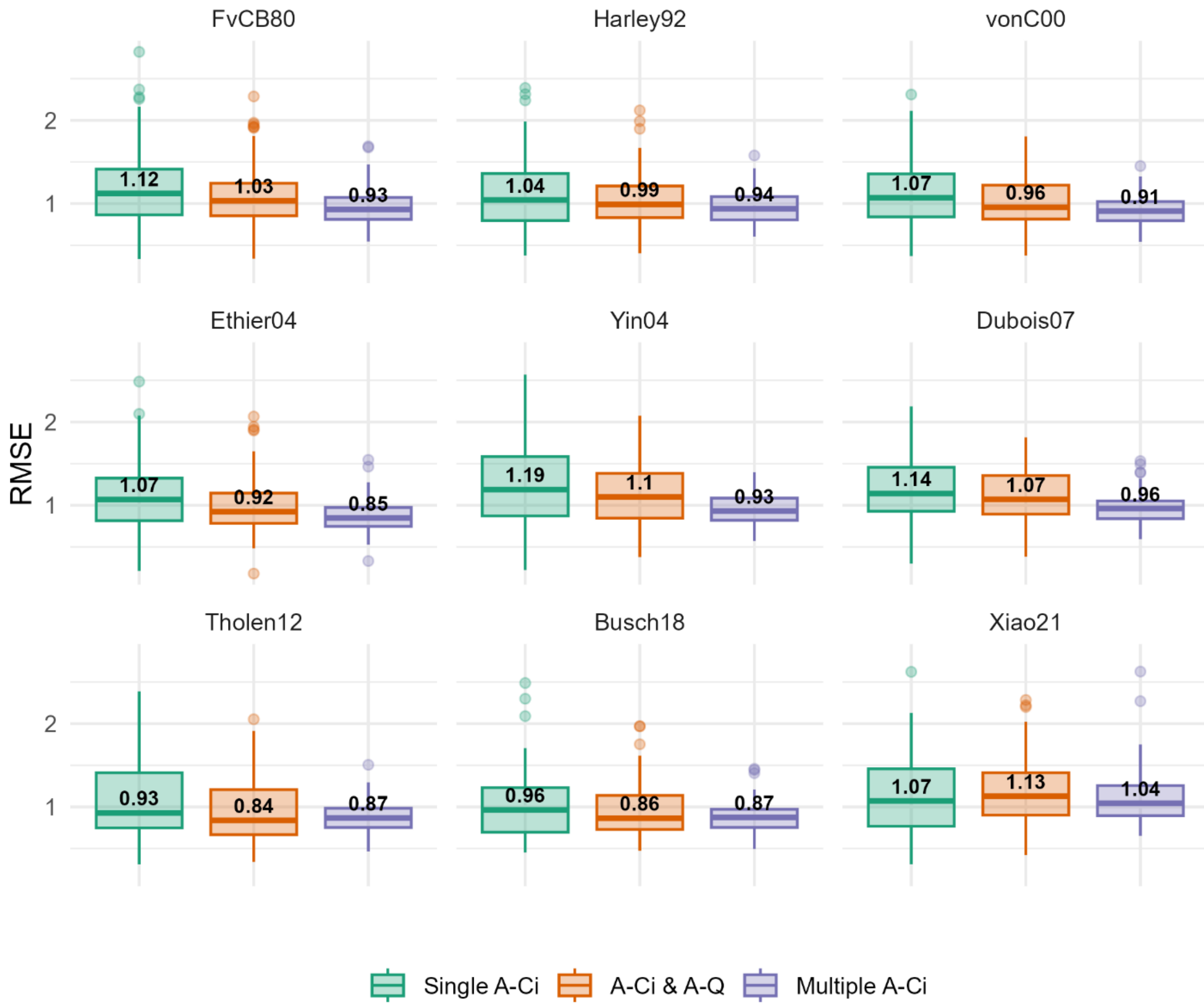

Supplementary Fig. S7. Overall RMSE of curve fitting across FvCB models and response curves. Boxplot shows the distribution of RMSE from Bayesian fits of simulated datasets using nine FvCB models. Colors indicate three types of response curves used for parameter estimation: A-Ci curves simulated at saturating light (1800 µmol m^{-2} s^{-1} PPFD, green), A-Ci curves at saturating light and A-Q curve at 300 µmol CO_2 mol^{-1} (orange), and A-Ci curves simulated at different light levels (purple). The numbers represent the median RMSE values across 100 simulated datasets.

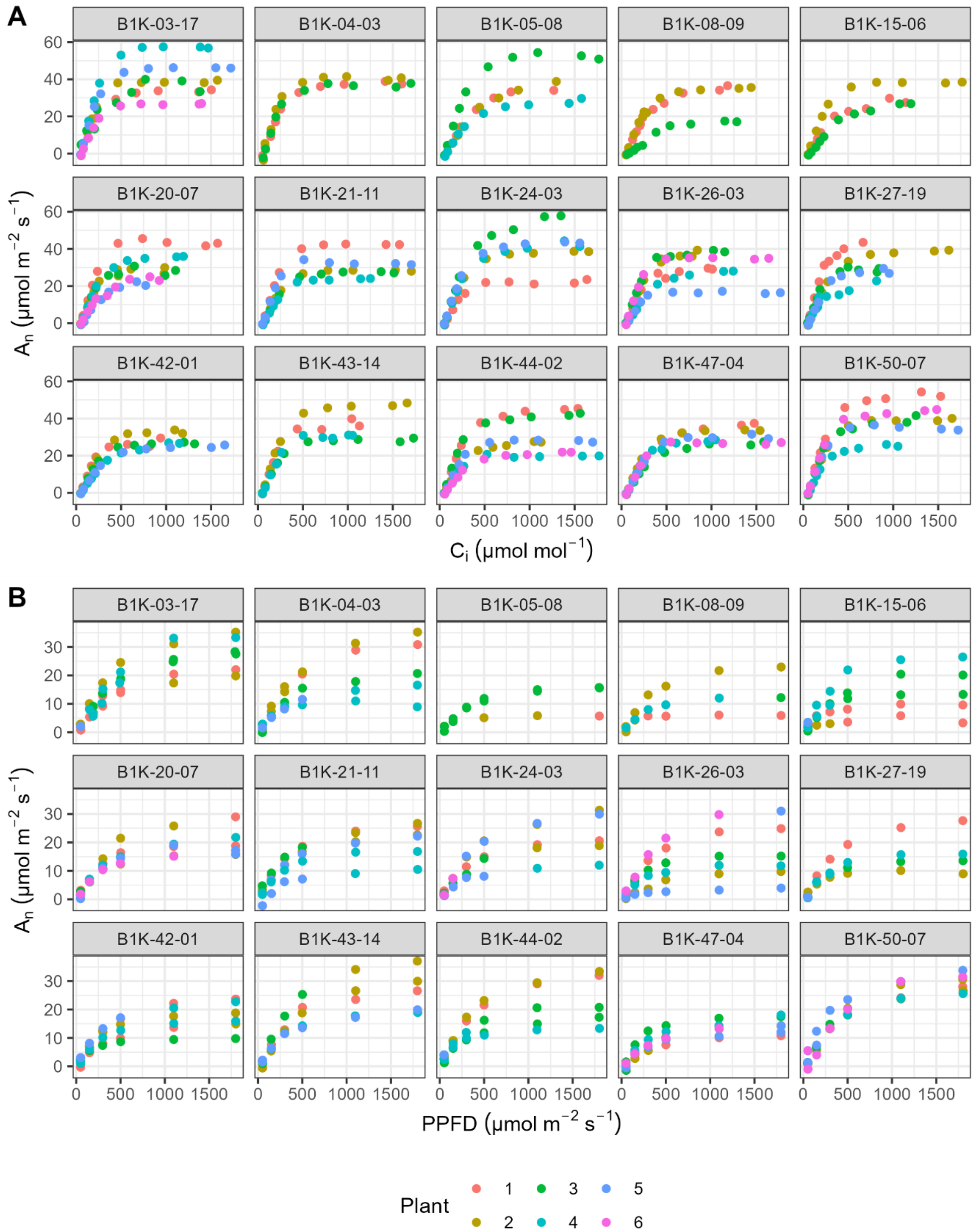

Supplementary Fig. S8. Distribution of measured A-Ci and A-Q response curves for the first 15 genotypes. (a) A-Ci curves measured for the first 15 genotypes out of a total of 85. Colors indicate individual plant-level measurement, with each genotype represented by two plots and up to three replicate plants per plot. (b) Corresponding A-Q curves measured for the same set of genotypes.

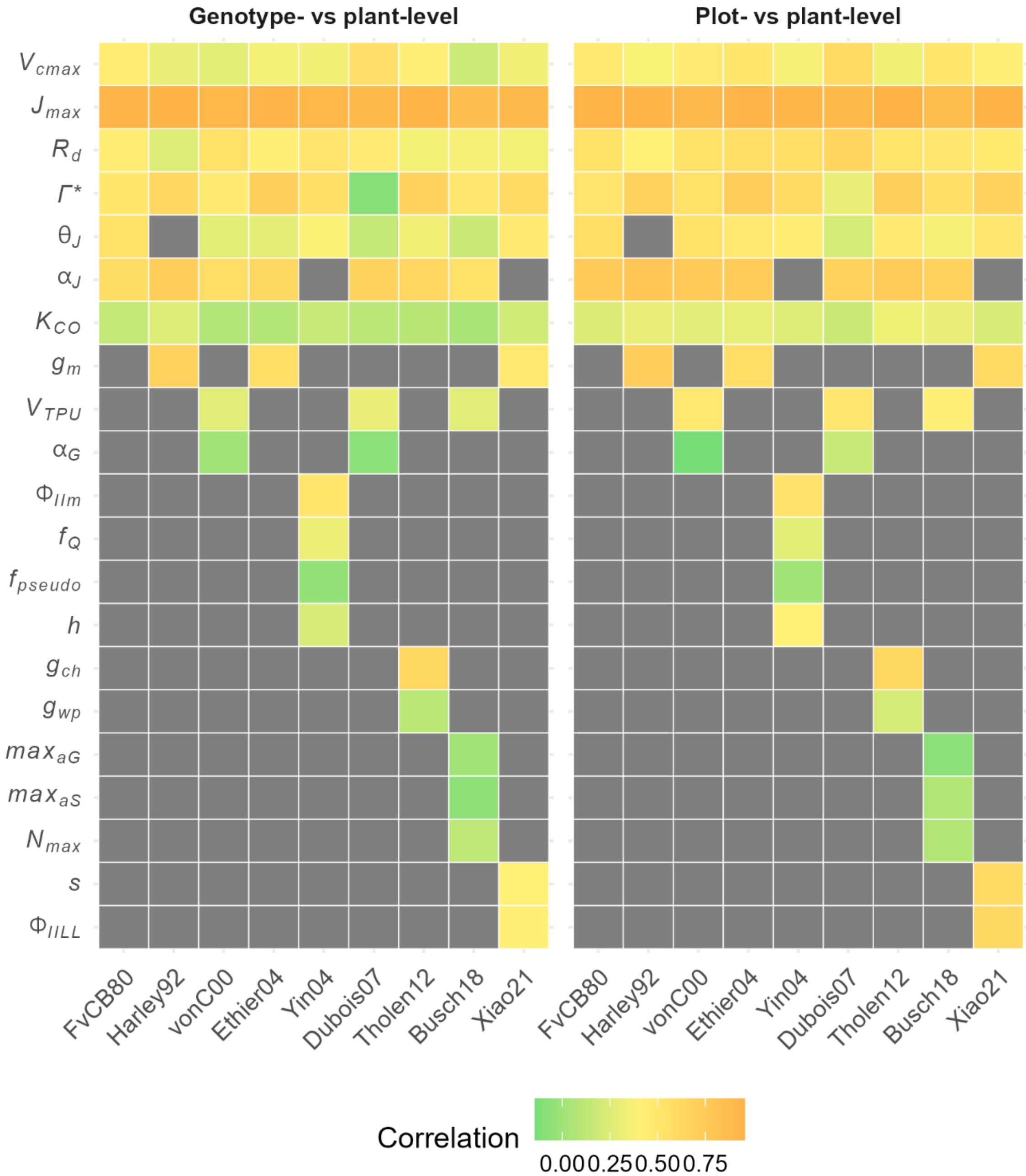

Supplementary Fig. S9. Consistency of parameter estimates across biological aggregation levels. Correlation between parameters estimated from plant-level data and those estimated from aggregated plot- or genotype-level data. For each model, parameter values obtained from individual plant fits were averaged to plot and genotype levels and compared with the corresponding estimates derived directly from plot- and genotype-level curve fitting.

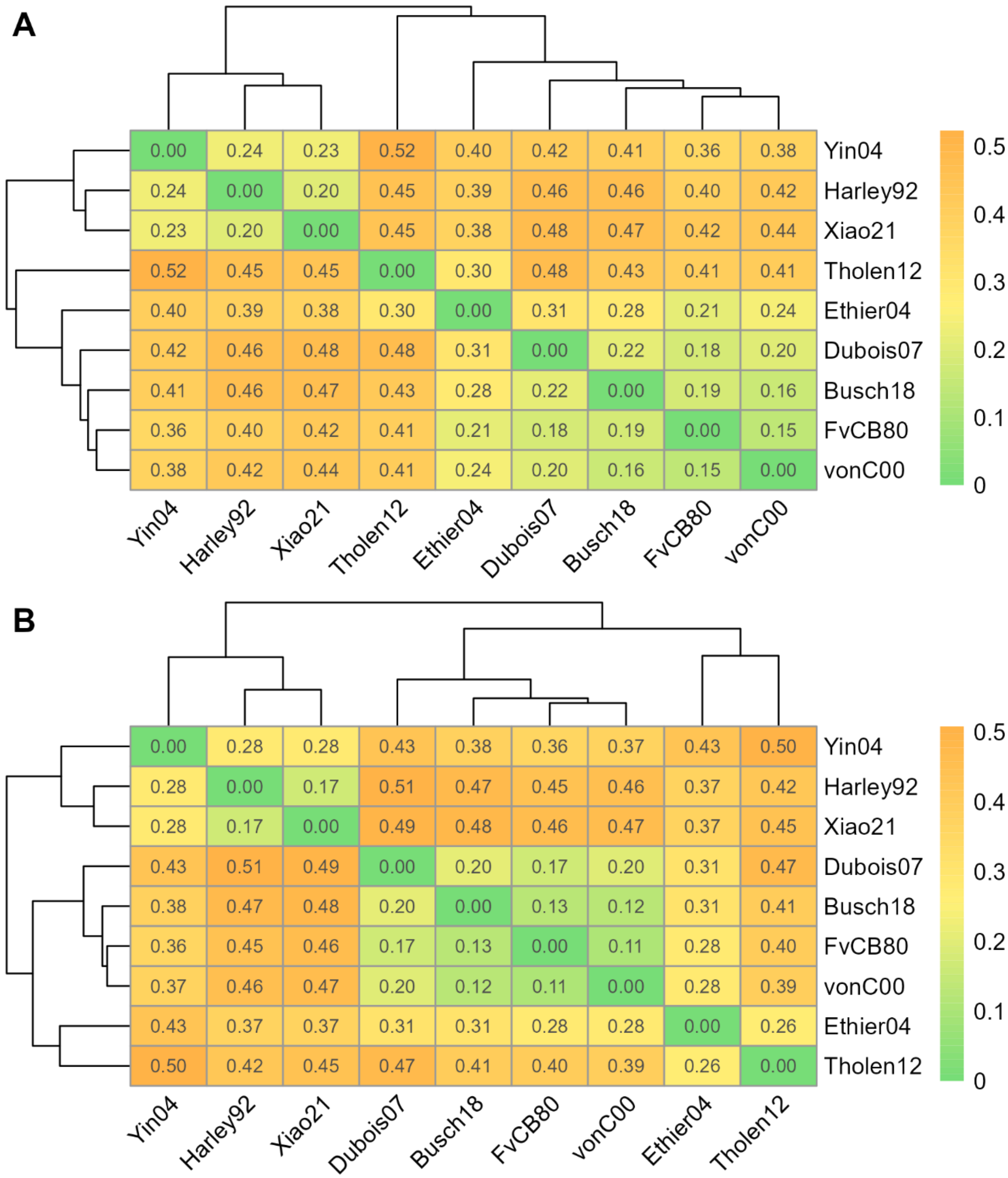

Supplementary Fig. S10. Dendrogram of nine FvCB models based on Euclidean distance. (a) Heatmap of mean Euclidean distance of parameter estimates between pairs of models across genotypes when A-Ci curves were applied for parameter estimation. The dendrogram illustrates the hierarchical clustering of models based on Euclidean distance. (b) Heatmap of mean Euclidean distance between models and dendrogram of model clustering when both A-Ci and A-Q curves were used.

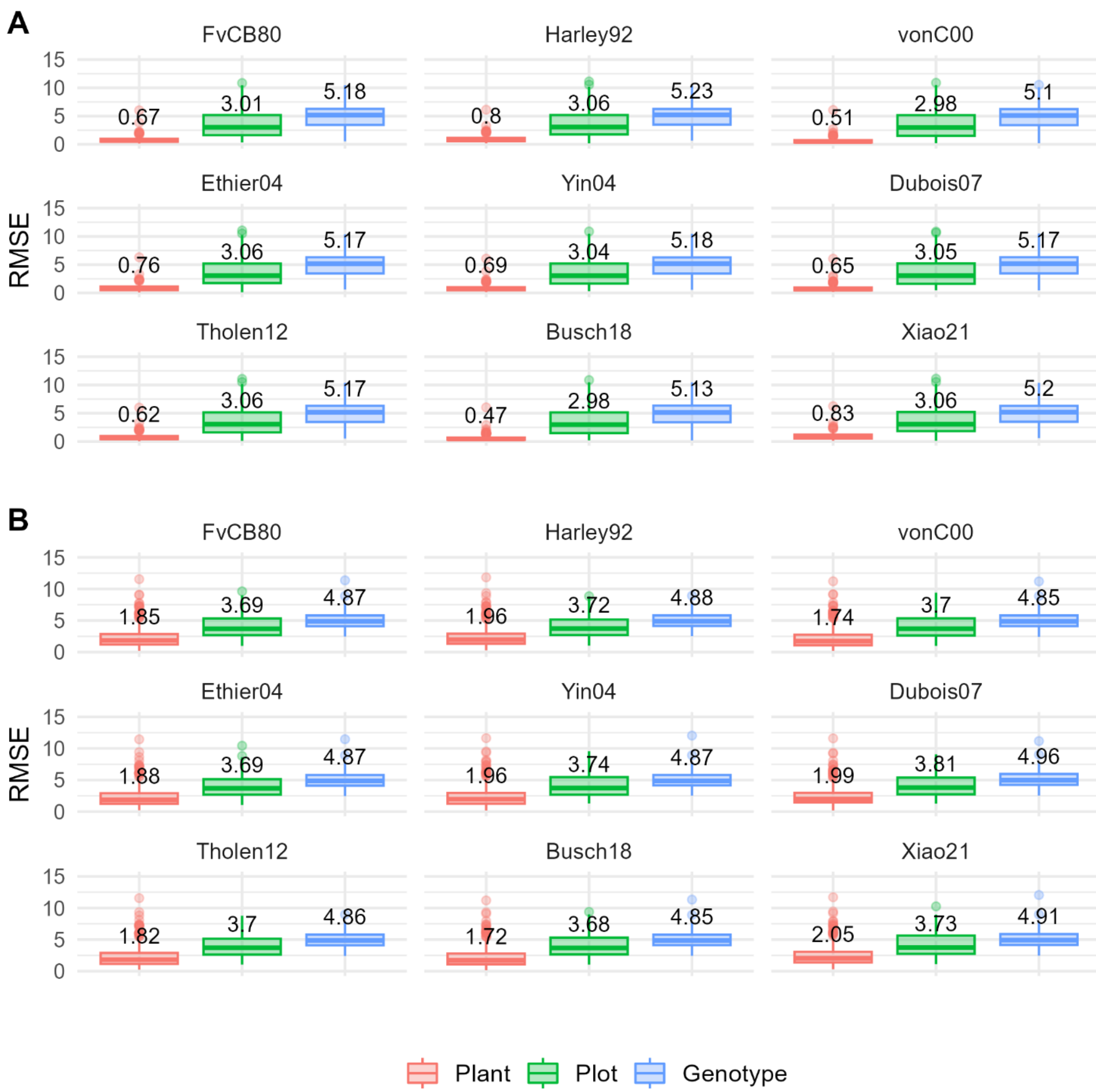

Supplementary Fig. S11. Summary of curve fitting accuracy across FvCB models using barley gas exchange data. (a) RMSE of nine models fitted to real A-Ci measurements at different levels: individual plant measurement (red), plot-level measurement (green) and genotype-level measurement (blue). (b) Corresponding RMSE values obtained when both A-Ci and A-Q curves were used for parameter estimation across the same three data levels.

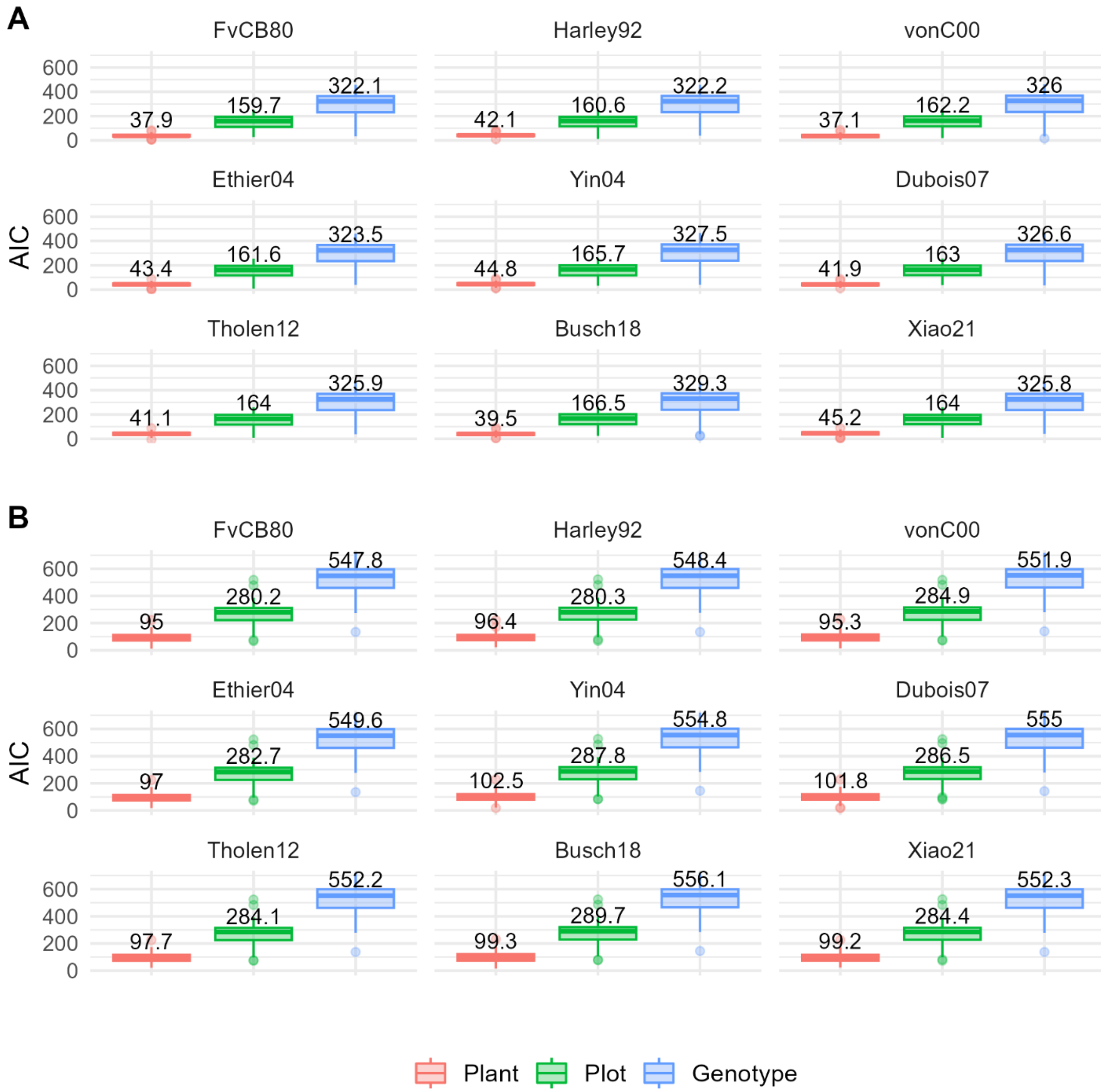

Supplementary Fig. S12. Selection of FvCB models using barley gas exchange data based on AIC score. (a) AIC score of nine models fitted to real A-Ci measurements at different levels: individual plant measurement (red), plot-level measurement (green) and genotype-level measurement (blue). (b) Corresponding AIC values obtained when both A-Ci and A-Q curves were used for parameter estimation across the same three data levels.
